# Sympathetic inputs regulate adaptive thermogenesis in brown adipose tissue through cAMP-Salt inducible kinase axis

**DOI:** 10.1038/s41598-018-29333-6

**Published:** 2018-07-20

**Authors:** Esther Paulo, Dongmei Wu, Yangmeng Wang, Yun Zhang, Yixuan Wu, Danielle L. Swaney, Margaret Soucheray, David Jimenez-Morales, Ajay Chawla, Nevan J. Krogan, Biao Wang

**Affiliations:** 1 Cardiovascular Research Institute, Department of Physiology, University of California, San Francisco, San Francisco, CA 94158 USA; 2 Department of Cellular and Molecular Pharmacology, University of California, San Francisco, San Francisco, CA 94158 USA; 3 California Institute for Quantitative Biosciences, QBI, University of California, San Francisco, San Francisco, CA 94158 USA; 4 J. David Gladstone Institutes, San Francisco, CA 94158 USA; 5 Present Address: Institute of Molecular Medicine, Peking-Tsinghua Center for Life Sciences, Peking University, 52 Haidian Road, Beijing, 100871 China; 6 Present Address: Department of Diabetes Complications and Metabolism, Beckman Research Institute of City of Hope, 1500 East Duarte Road, Duarte, CA 91010 USA

## Abstract

Various physiological stimuli, such as cold environment, diet, and hormones, trigger brown adipose tissue (BAT) to produce heat through sympathetic nervous system (SNS)- and β-adrenergic receptors (βARs). The βAR stimulation increases intracellular cAMP levels through heterotrimeric G proteins and adenylate cyclases, but the processes by which cAMP modulates brown adipocyte function are not fully understood. Here we described that specific ablation of cAMP production in brown adipocytes led to reduced lipolysis, mitochondrial biogenesis, uncoupling protein 1 (Ucp1) expression, and consequently defective adaptive thermogenesis. Elevated cAMP signaling by sympathetic activation inhibited Salt-inducible kinase 2 (Sik2) through protein kinase A (PKA)-mediated phosphorylation in brown adipose tissue. Inhibition of SIKs enhanced Ucp1 expression in differentiated brown adipocytes and Sik2 knockout mice exhibited enhanced adaptive thermogenesis at thermoneutrality in an Ucp1-dependent manner. Taken together, our data indicate that suppressing Sik2 by PKA-mediated phosphorylation is a requisite for SNS-induced Ucp1 expression and adaptive thermogenesis in BAT, and targeting Sik2 may present a novel therapeutic strategy to ramp up BAT thermogenic activity in humans.

## Introduction

Energy balance requires equivalent energy intake and energy expenditure, and when energy intake exceeds energy expenditure, animals store excess energy as fat in adipose and other metabolic tissues. Chronic energy excess can lead to obesity and further development into type II diabetes^1,2^. Whereas most current drug-based therapies for obesity mainly aim to reduce total energy intake using appetite suppressants and nutrient-absorption inhibitors, increasing energy expenditure presents a good alternative^3–5^. Adaptive thermogenesis refers to body heat production in response to environmental changes. It occurs in brown fat^6,7^, which contains specialized mitochondria-rich and uncoupling protein 1 (UCP1) positive brown adipocytes^8–10^. The importance of brown fat-dependent thermoregulation has been demonstrated in rodents. For example, genetic ablation of BAT leads to defective thermoregulation and obesity^11,12^, while BAT transplant improves whole-body energy metabolism in mice^13–15^. Human brown fat activity gradually declines with aging and metabolic diseases such as obesity and diabetes^16–18^. Because human brown fat may contribute significantly to total energy expenditure (300 kcal more a day if fully stimulated)^19,20^, increasing brown fat-dependent adaptive thermogenesis could potentially reduce adiposity and improve metabolic health^21^.

Cold exposure can activate adaptive thermogenesis in BAT through sympathetic nervous system (SNS)-dependent activation of β-adrenergic receptors (βARs)^22,23^. The β1-AR is critical for brown adipocyte proliferation^24^, while adipocyte-specific β3-AR is required for thermogenic activation on mature brown adipocytes^25–27^. Mice deficient of all three βARs, the βless mice, exhibit impaired cold- and diet-induced thermogenesis, and they are obese and show signs of insulin resistance and hepatic steatosis^23^, highlighting the importance of βAR signaling in adaptive thermogenesis in the setting of global βAR deficiency. The β3-AR activation is also crucial for the development of beige adipocytes, which are another UCP1-positive adipocytes formed and clustered within the subcutaneous white adipose tissue (WAT). Systemic β3-AR activation in rodents increases thermogenic capacities of both brown and beige adipocytes, reduces total fat mass and improves metabolic performance^28^. Agonists of β3-AR have been tested as insulin sensitization and anti-obesity drugs in humans, but with limited success^29–31^. It is possible that the differences between the mouse and human β3-AR proteins may explain these failures; a thorough characterization of βAR signaling mechanisms in brown (and beige) fat may provide new druggable targets to ramp up thermogenic activity.

The βAR stimulation elevates intracellular cAMP production through the guanine nucleotide-binding alpha stimulating protein (Gnas), the stimulatory G-protein alpha subunit. Elevated cAMP levels activate protein kinase A (PKA), which in turn phosphorylates cAMP-responsive element binding protein (CREB) in a characteristic “burst-attenuation-refractory” fashion^32^. Elevations of cAMP in cells activate PKA, which in turn phosphorylates and inhibits Salt-inducible kinase 2 (Sik2), a member of AMPK-related kinase family^33^. We and others have previously demonstrated that SIKs are regulated by cAMP signaling in fatbody in *Drosophila* and hepatocytes in mammals^32,34–36^. We have investigated physiological impacts of SIK deficiency on beige adipocyte formation *in vivo*^37^. Since cAMP signaling regulates multiple steps of adaptive thermogenesis in BAT, including lipolysis, mitochondrial biogenesis and Ucp1 expression, the physiological roles of SIKs, Sik2 in particular, in adaptive thermogenesis in brown adipocytes have not been fully investigated. Here we showed that brown adipocyte-specific adenylate cyclase-stimulating G alpha (Gnas) knockout mice, the Gnas^BKO^ mice, exhibited reduced Ucp1 expression and mitochondrial biogenesis in BAT and defective adaptive thermogenesis. Elevations of intracellular cAMP in response to cold stimulation inhibited Sik2, and Sik2 knockout mice showed enhanced Ucp1 expression and increased thermogenic capacity housed at thermoneutrality. Collectively, our study demonstrates a novel regulatory mechanism of adaptive thermogenesis in the BAT.

## Results

### The cAMP production in brown adipocytes is required for cold-induced adaptive thermogenesis

**Figure 1:**
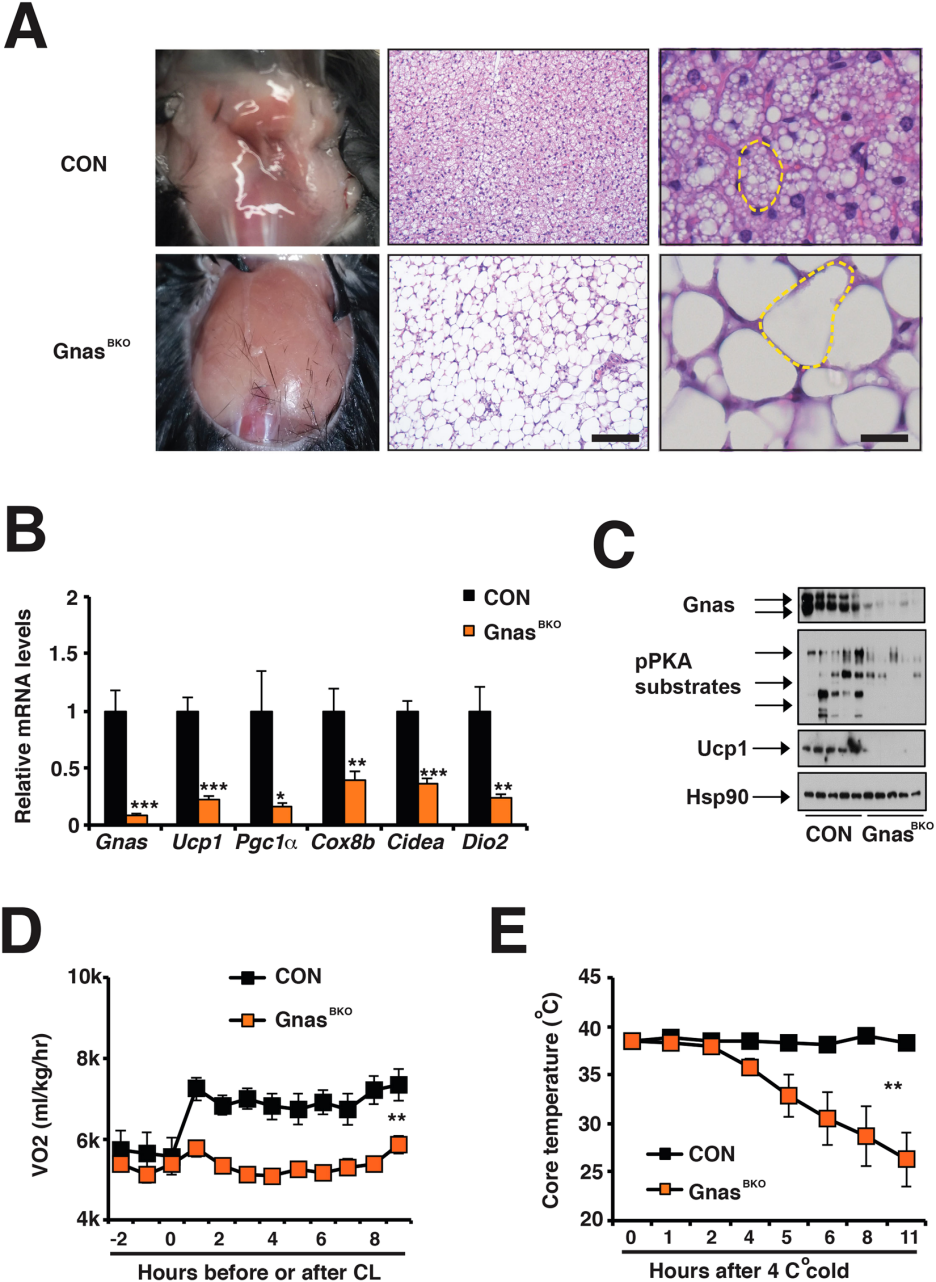
Gnas^BKO^ mice showed reduced Ucp1 expression in iBAT and defective adaptive thermogenesis. (**A**) Gross view and H&E staining of dissected iBAT in ~6–8-week-old male CON and Gnas^BKO^ mice. Scale bar: 200 μm. A single adipocyte was outlined by a dashed yellow line. Scale bar: 50 μm. (**B**) q-PCR analysis of *Gnas*, *Ucp1, Pgc1α, Cox8b, Cidea* and *Dio2* mRNA levels in iBAT from 6–8 week-old male CON and Gnas^BKO^ mice. Sample sizes: n = 7 for both genotypes. (**C**) Immunoblots showing amounts of Gnas, phosphor-PKA substrates, Ucp1 and Hsp90 in iBAT from 6–8 week-old male CON and Gnas^BKO^ mice. (**D**) Oxygen consumption recordings in response to CL in 6–8 week-old male CON and Gnas^BKO^ mice. Sample size: CON (n = 4) and Gnas^BKO^ (n = 3). (**E**) Core temperature of 6–8 –week-old male CON and Gnas^BKO^ mice upon 4 °C cold challenge. Sample size: CON (n = 12) and Gnas^BKO^ (n = 9).

In order to investigate effects of βAR-cAMP signaling deficiency in brown adipocytes, we have generated Ucp1-Cre;Gnas^f/f^ (Gnas^BKO^) mice. Cre-negative Gnas^f/f^ mice were used as controls. Gnas mRNA and protein levels were specifically reduced in interscapular brown adipose tissue (iBAT), but not in other tissues (Supplementary Fig. 1, Fig. 1B–C). Protein kinase A (PKA) is activated upon elevated cAMP levels. Consistently, PKA activity (determined by immunoblot of phosphor-PKA substrate antibody) was abolished in the iBAT of Gnas^BKO^ mice (Fig. 1C). Similar to βless mice^23^, Gnas^BKO^ mice had a pale and enlarged iBAT (Fig. 1A). Their brown adipocytes exhibited white adipocyte-like morphology, containing a single and large lipid droplet (Fig. 1A). They also showed reduced thermogenic gene expression, such as *Ucp1, Pgc1α, Dio2, Cox8b*, and *Cidea* (Fig. 1B). Ucp1 protein levels were also diminished in iBAT of Gnas^BKO^ mice (Fig. 1C). We further examined the thermogenic capacity in Gnas^BKO^ mice. In the indirect calorimetry experiment, murine-selective β3-AR agonist CL-316,243 (CL) failed to induce oxygen consumption in Gnas^BKO^ mice (Fig. 1D), even though there were no differences in basal O2 consumption, respiratory exchange ratio (RER), food intake and physical activity (Supplementary Fig. 2A–E). Consequently, Gnas^BKO^ mice could not maintain their core temperature under 4 °C cold challenge (Fig. 1E). Thus, cAMP signaling in brown adipocytes is required for BAT thermogenic function.

**Figure 2:**
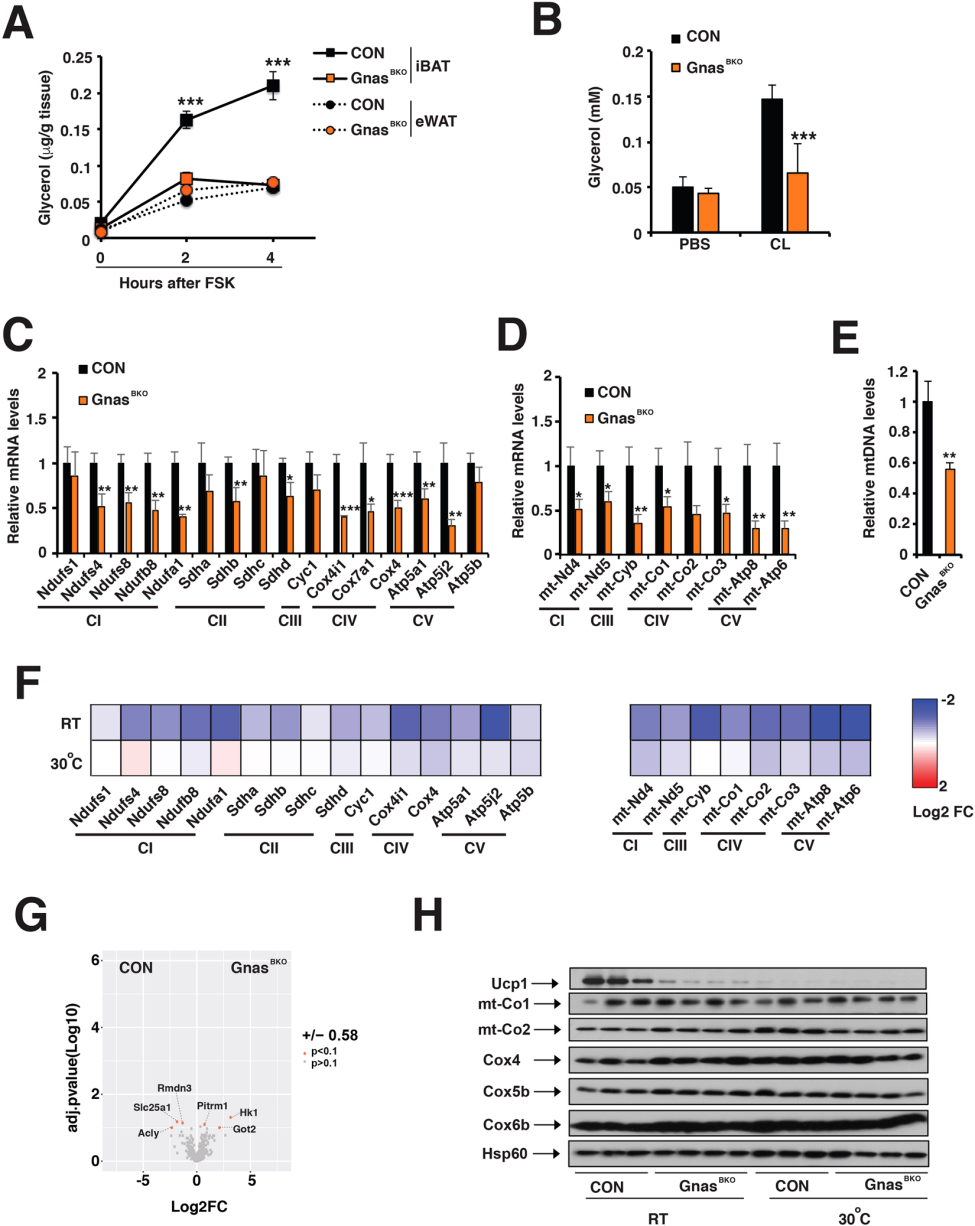
Gnas^BKO^ mice exhibited attenuated lipolysis and mitochondrial biogenesis in iBAT. (**A**) Glycerol released *in vitro* prior to and after Forskolin (10 uM FSK) stimulation from iBAT and eWAT from 6–8-week-old CON and Gnas^BKO^ mice. Sample size: n = 10 per genotypes. (**B**) Serum glycerol levels prior to and one-hour after CL injection in 6–8-week-old CON and Gnas^BKO^ mice. Sample size: CON (n = 5) and Gnas^BKO^ (n = 4). q-PCR analysis of relative mRNA levels of nuclear (**C**) and mitochondrial (**D**) encoded ETC gene expression in 6–8-week-old CON and Gnas^BKO^ mice. Sample size: n = 5 per genotypes. (**E**) Relative mitochondrial DNA (mtDNA) levels in the iBAT of 6–8-week-old CON and Gnas^BKO^ mice. Sample size: n = 5 per genotypes. (**F**) Heatmap showing log2 fold changes of mRNA levels of nuclear and mitochondrial encoded ETC gene expression in 6–8-week-old CON and Gnas^BKO^ mice housed at room temperature (RT) and thermoneutrality (30 °C). (**G**) Volcano plots showing significantly (p < 0.1) down- or up-regulated mitochondrial proteins (over 1.5 fold) in isolated iBAT mitochondria from 6–8-week-old male Gnas^BKO^ mice. (**H**) Immunoblots showing amounts of Ucp1, C-IV subunits (mt-Co1, mt-Co2, Cox4, Cox5b, and Cox6b1) and Hsp60 in iBAT mitochondria from 6–8-week-old male CON and Gnas^BKO^ mice housed at RT and 30 °C.

Besides Ucp1-mediated proton leak, adaptive thermogenesis also requires lipolysis to generate fatty acid and mitochondrial respiration to generate proton gradient across the mitochondrial membrane. Indeed, Gnas^BKO^ mice exhibited reduced *in vitro* Forskolin (FSK)-induced lipolytic activity of iBAT (Fig. 2A), which was consistent with reduced PKA activity (Fig. 1C). The *in vitro* FSK-induced lipolytic activity in epididymal WAT (eWAT) was not altered in Gnas^BKO^ mice (Fig. 2A). Additionally, CL-induced serum glycerol levels were lower in Gnas^BKO^ mice, showing an attenuated lipolytic response *in vivo* (Fig. 2B). The cAMP signaling in brown adipocytes drives mitochondrial biogenesis through promoting transcription of the Peroxisome proliferator-activator gamma coactivator 1 alpha (Pgc1α); *Pgc1α* mRNA was reduced in the iBAT of Gnas^BKO^ mice (Fig. 1B). Consistently, we observed reduced expression of most mitochondrial ETC genes encoded by both nuclear and mitochondrial genomes (Fig. 2.C,D). Consistently, the mitochondrial DNA copy numbers were reduced by half in the iBAT of Gnas^BKO^ mice (Fig. 2E). We further performed mass spectrometry analysis of isolated mitochondria from iBAT of control and Gnas^BKO^ mice^38,39^. We identified more than 630 mitochondrial proteins (roughly 60% of the mitochondrial proteins listed in MitoCarta2.0) (Supplementary Fig. 3A). However, mitochondrial proteome in isolated iBAT mitochondria was minimally affected by Gnas deficiency (Fig. 2G). Hexokinase 1 (Hk1) was the most upregulated protein in iBAT mitochondria from Gnas^BKO^ mice in the mass spectrometry dataset, which was confirmed by immunoblots (Supplementary Fig. 3B). Therefore, despite reduced mRNA levels of electron transport chain (ETC) subunits, the ETC proteome composition was not affected in iBAT of Gnas^BKO^ mice. For example, the complex IV protein levels (mtDNA-encoded mt-Co1 and mt-Co2, nuclear-encoded Cox4, Cox5b, and Cox6b) in isolated mitochondria were not affected by Gnas deficiency (Fig. 2H). This data suggests that cAMP signaling controls lipolysis (though PKA activation) and mitochondrial biogenesis (through regulating *Pgc1α* transcription) in brown adipose tissue.

Animals at thermal neutral zone (~30 °C for mice) maintain their body temperature through its basal metabolism without additional thermogenesis and physical activity. There is minimal sympathetic flow to BAT for mice housed at thermoneutrality (~30 °C). However, housing mice at room temperature (RT, ~22 °C) results in the activation of the BAT-mediated adaptive thermogenesis. Interestingly, reductions in mRNA levels of ETC subunits and Ucp1 proteins in iBAT were less profound in Gnas^BKO^ mice housed at thermoneutrality (Fig. 2F,H), suggesting that sympathetic inputs regulate *Ucp1* and *Pgc1α* transcription through Gnas-mediated cAMP generation in brown adipocytes.

### The cAMP production in beige adipocytes is dispensable for cold-induced beige adipocyte renaissance

SNS-induced βAR activation is also critical for the formation of beige adipocytes, brown-like adipocytes with multilocular morphology and Ucp1-dependent thermogenic activity^40,41^. For example, cold failed to induce beige adipocyte formation in adult βless mice (Supplementary Fig. 4D), highlighting the importance of βAR signaling in beige adipocyte formation *in vivo*. Additionally, chemical denervation with 6-hydroxydopamine (6-OHDA) prior to 3-week of age, or prior to 7-day 8 °C cold challenge in adult mice led to reduced *Ucp1* mRNA expression (Supplementary Fig. 4A). Western blot confirmed that 6-OHDA suppressed PKA activity and Ucp1 protein levels in 3-week-old pups and cold-treated adult mice (Supplementary Fig. 4B,C), suggesting that sympathetic innervation was required for beige adipocyte genesis during postnatal development and cold-induced beige adipocyte formation in adult mice.

To address whether cAMP in beige adipocytes themselves is necessary for beige adipocyte formation in iWAT, we compared *Ucp1* expression and beige adipocyte abundance in iWAT in Gnas^BKO^ and adipocyte-specific Gnas knockout mice (Adiponectin-Cre;Gnas^f/f^; Gnas^AKO^). The postnatal beige adipocyte developed normally in iWAT from 3-week-old Gnas^BKO^ and Gnas^AKO^ pups, indicating that adipocyte cAMP signaling was dispensable for *de novo* beige adipocyte formation during postnatal development (Supplementary Fig. 4E). However, 7-day 8 °C cold treatment robustly induced *Ucp1* transcription in iWAT of adult Gnas^BKO^ and control mice, but had no effect in Gnas^AKO^ mice (Supplementary Fig. 4E). Histology analysis confirmed that multilocular beige adipocytes reappeared in iWAT of cold-treated Gnas^BKO^ mice (Supplementary Fig. 4F), demonstrating that cAMP signaling in beige adipocytes themselves was dispensable for their maintenance *in vivo* at adult stage (Supplementary Fig. 4G).

### Gnas^BKO^ mice are not obese under HFD despite thermogenic defects in iBAT

**Figure 3:**
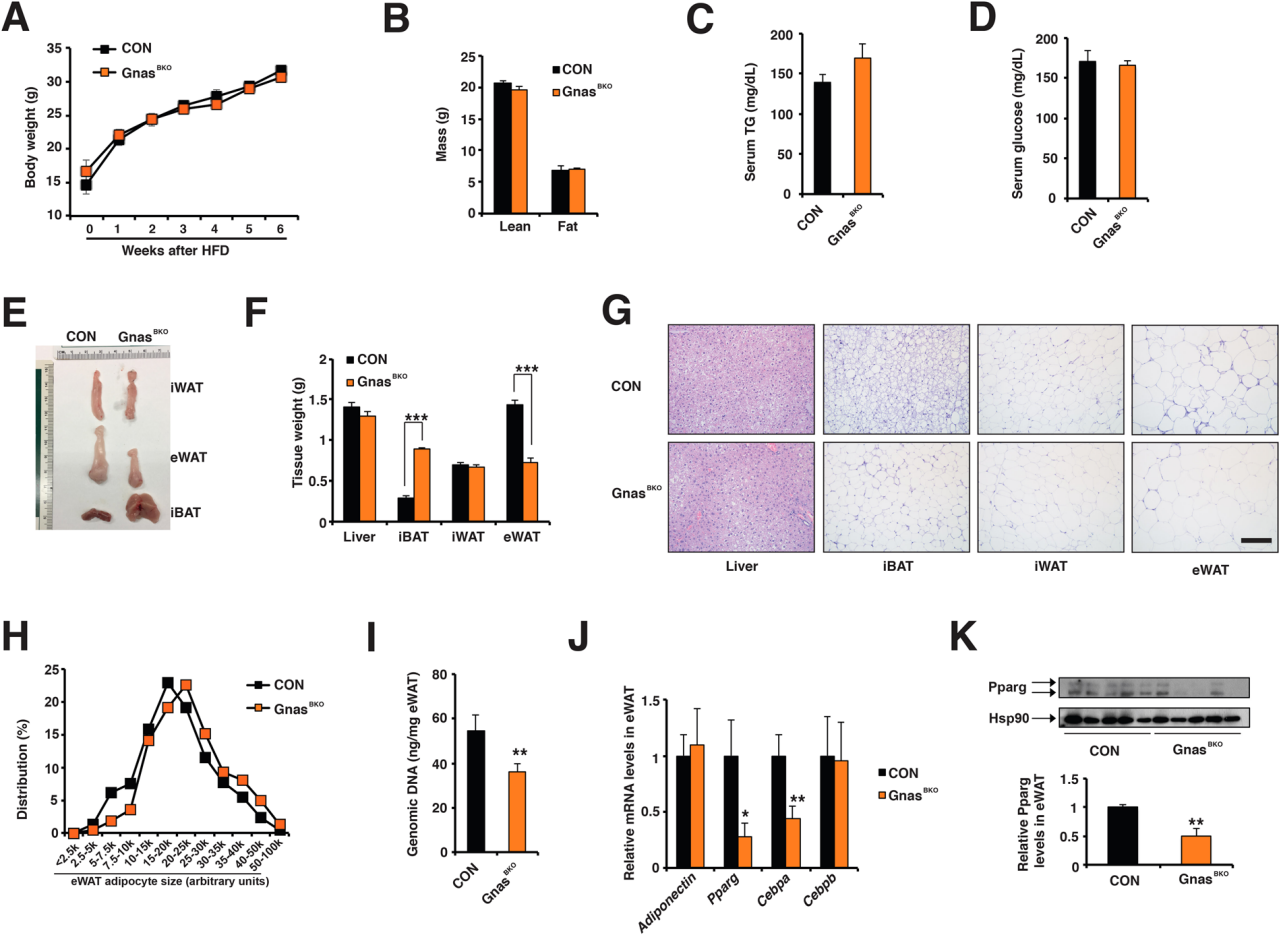
Gnas^BKO^ mice were not protected from HFD-induced obesity. (**A**) Body weight in male CON and Gnas^BKO^ mice upon 6-week HFD (started at ~5–6-week of age). Sample size: CON (n = 11) and Gnas^BKO^ (n = 14). (**B**) DEXA measurements of CON and Gnas^BKO^ mice after HFD. Sample size: CON (n = 6) and Gnas^BKO^ (n = 9). Levels of fasting serum TG (**C**) and glucose (**D**) in CON and Gnas^BKO^ mice after HFD. Sample size: CON (n = 8) and Gnas^BKO^ (n = 11). (**E**) Representative images of iWAT, eWAT and iBAT from CON and Gnas^BKO^ mice after HFD. (**F**) Tissue mass of iWAT, eWAT, BAT and liver from CON and Gnas^BKO^ mice after HFD. Sample size: CON (n = 6) and Gnas^BKO^ (n = 9). (**G**) Representative H&E staining of liver, iBAT, iWAT and eWAT from CON and Gnas^BKO^ mice after HFD. Scale bar: 100 μm. (**H**) Adipocyte size distribution in the eWAT from CON and Gnas^BKO^ mice after HFD. Total adipocytes counted: CON (n = 520) and Gnas^BKO^ (n = 488). (**I**) Genomic DNA content per eWAT weight in CON and Gnas^BKO^ mice after HFD. Sample size: n = 6 for both genotypes. (**J**) q-PCR analysis of *Adiponectin*, *Pparg, Cebpa* and *Cebpb* mRNA levels in the eWAT from CON and Gnas^BKO^ mice after HFD. Sample sizes: n = 11 for both genotypes. (**K**) Immunoblots showing amounts of Pparg in the eWAT from CON and Gnas^BKO^ mice after HFD. Quantifications of Pparg immunoblots showed below.

We then accessed whether defective adaptive thermogenesis in BAT was linked to metabolic dysfunctions in Gnas^BKO^ mice. At room temperature (RT), the Gnas^BKO^ mice had normal body weight, lean and fat mass under normal chow feeding, their visceral fat mass was specifically reduced at the expense of enlarged iBAT (Supplementary Fig. 5A–B). This fat redistribution was not due to a secondary adaptive response triggered by the defective adaptive thermogenesis, because it was also present in Gnas^BKO^ mice housed at thermoneutrality (Supplementary Fig. 5B). After 6-week high-fat diet (HFD), the Gnas^BKO^ mice showed no differences in body weight, and lean and fat mass (Fig. 3A–B), and levels of fasting serum TG and glucose remained unchanged (Fig. 3C–D). The iBAT in Gnas^BKO^ mice had a three-fold increase in size and contained unilocular lipid-filled adipocytes (Fig. 3E–G). In contrast, their eWAT mass was reduced by half (Fig. 3E–G), although their lipolytic activity or adipocyte size was not altered (Fig. 3G–H, Fig. 2A). The genomic content in the eWAT was reduced in the Gnas^BKO^ mice (Fig. 3I), suggesting that reduction of adipocyte numbers may account for smaller eWAT mass in the Gnas^BKO^ mice. Pro-adipogenic *Cebpa* and *Pparg* gene expressions (and Pparg protein levels) were diminished in the eWAT of Gnas^BKO^ mice, without the change of the abundance of Pdgfra + Sca1 + progenitors (Fig. 3J–K, Supplementary Fig. 6). Notably, the fat redistribution between iBAT and eWAT was not observed in Gnas^AKO^ mice^42^. Collectively, diminished thermogenic capacity in Gnas^BKO^ mice was not associated with significant metabolic abnormalities under normal chow and HFD.

### The cAMP signaling inhibits Salt-inducible kinases (SIKs) in BAT in response to sympathetic inputs

**Figure 4:**
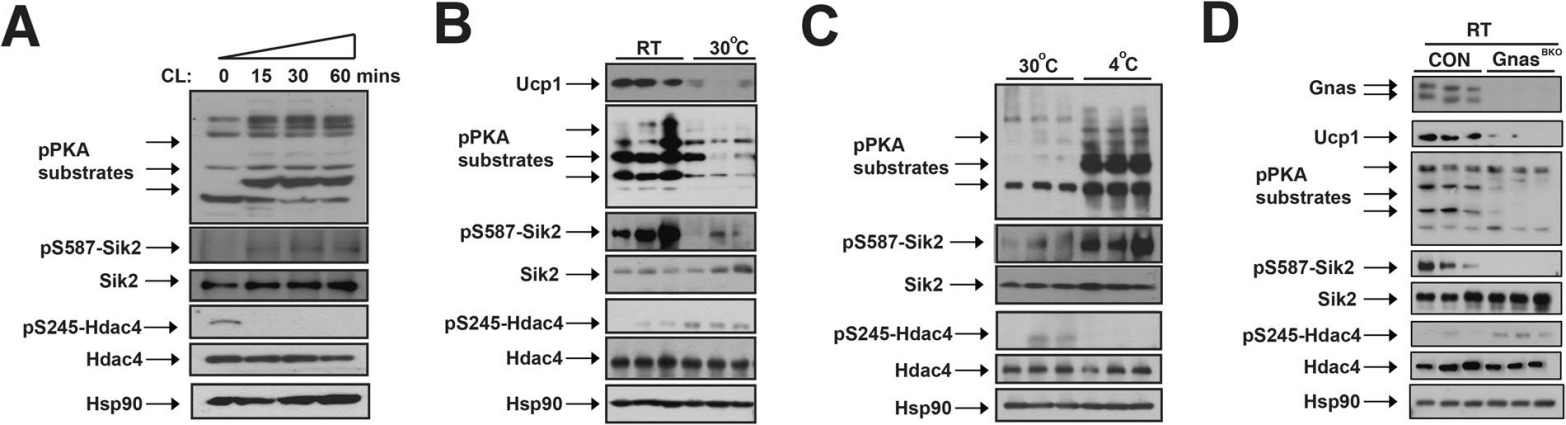
Cold inactivated Salt-inducible kinase 2 (Sik2) in iBAT through cAMP production. (**A**) Immunoblots showing amounts of pS587 and total Sik2, pS245 and total Hdac4, pS563 and total Hsl, and Hsp90 in differentiated brown adipocytes. CL time-course shown. (**B**) Immunoblots showing amounts of Ucp1, phosphor-PKA substrates, pS587 and total Sik2, pS245 and total Hdac4, and Hsp90 in iBAT from ~10-week-old male C57bl/6 J mice housed at RT and thermoneutrality. (**C**) Immunoblots showing amounts of phosphor-PKA substrates, pS587 and total Sik2, pS245 and total Hdac4, and Hsp90 in iBAT from ~10-week-old male C57bl/6 J mice housed at thermoneutrality and after 30-minute 4 °C cold stimulation. (**D**) Immunoblots showing amounts of Gnas, Ucp1, phosphor-PKA substrates, pS587 and total Sik2, pS245 and total Hdac4, and Hsp90 in iBAT from ~6–8-week-old male CON and Gnas^BKO^ mice housed at RT.

Mammalian SIK family contains three members: Sik1, 2 and 3^43–45^. Both Sik1 and Sik2 are expressed in mature adipocytes compared to stromal-vascular fraction (SVF) cells, although Sik2 is an adipose-enriched SIK isoform, which is abundantly expressed in many fat depots (Supplementary Fig. 7)^37,45^. To test whether sympathetic nerves regulate SIK activity and whether this regulation is required for thermogenic gene expression, we analyzed CL’s effect on Sik2 activity in differentiated brown adipocyte. We had previously shown that Sik2 S587 phosphorylation is a negative indicator of its kinase activity, because hyper-phosphorylated Sik2 was accompanied with de-phosphorylation of its known substrates (CRTCs and HDAC4)^32,34^. In *in vitro* differentiated brown adipocytes, CL treatment robustly induced PKA signaling (Fig. 4A). Sik2 was hyper-phosphorylated at Ser587 in response to CL in differentiated brown adipocytes, and consequently, its substrate Hdac4 was hypo-phosphorylated (Fig. 4A). These data suggested that Sik2 activity was inhibited by cAMP-PKA signaling in brown adipocytes *in vitro*.

We then determined whether Sik2 activity in brown adipose tissue was differentially regulated at RT and thermoneutrality *in vivo*. Ucp1 expression and PKA activity were reduced at thermoneutrality in iBAT from C57bl/6 J mice due to reduced sympathetic inputs (Fig. 4B). Along with reduced PKA activity, Sik2 was hypo-phosphorylated at Ser587 and Hdac4 was hyper-phosphorylated at Ser245 in iBAT at thermoneutrality (Fig. 4B). On the other hand, acute 4 °C cold stimulation for half hour robustly increased cAMP signaling in iBAT, which led to Sik2 Ser587 hyper-phosphorylation and Hdac4 Ser245 hypo-phosphorylation (Fig. 4C). The Gnas^BKO^ mice had no PKA signaling in iBAT (Fig. 1C), hence, Sik2 was hypo-phosphorylated and active in the iBAT of Gnas^BKO^ mice at RT (Fig. 4D). Collectively, we conclude that Sik2 activity is negatively correlated with sympathetic activity and Ucp1 expression in iBAT.

### SIKs inhibits thermogenic gene expression in brown adipocytes *in vitro* and *in vivo*

**Figure 5:**
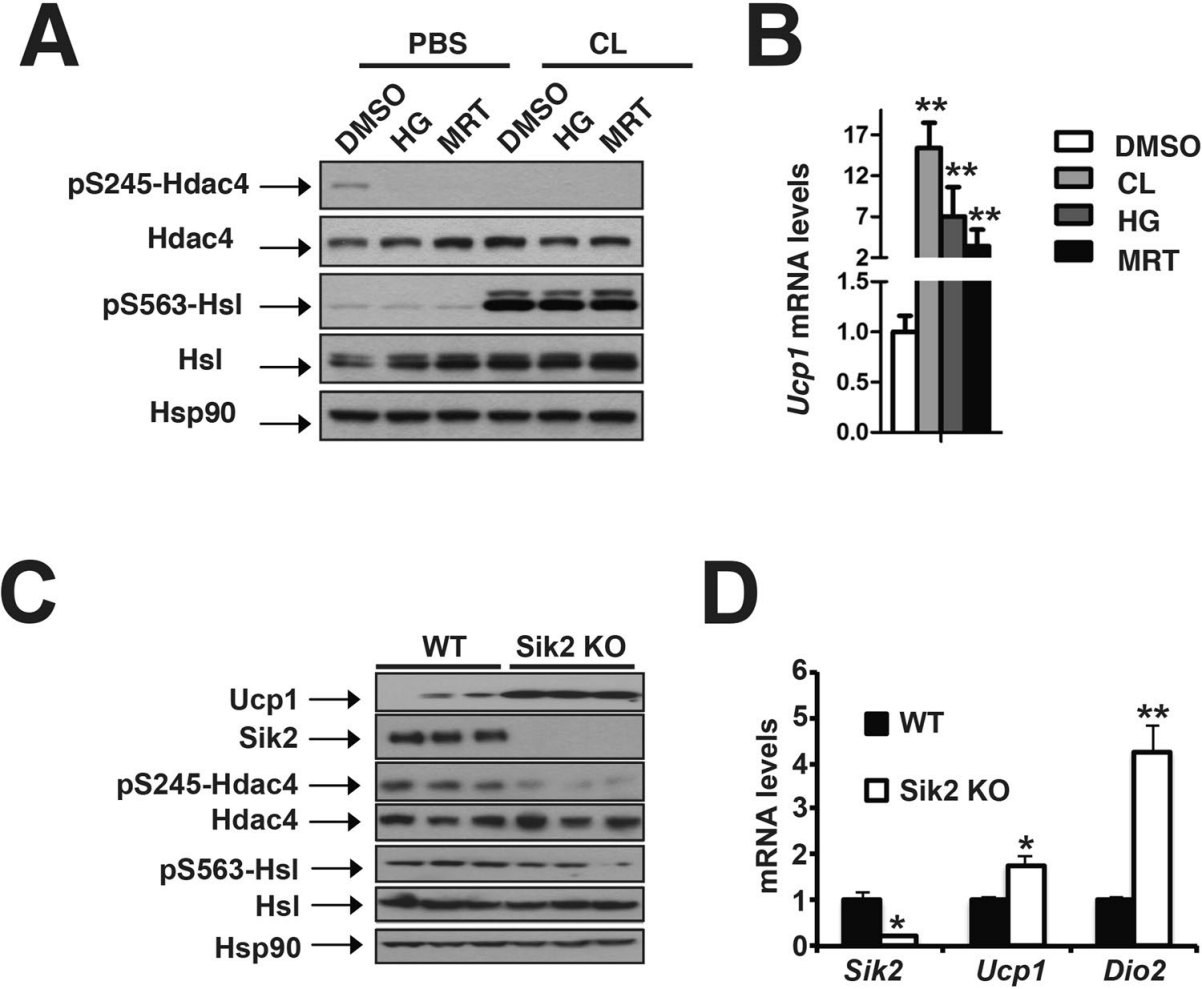
Sik2 suppressed thermogenic gene expression. (**A**) Immunoblots showing amounts of pS245 and total Hdac4, pS563 and total Hsl, and Hsp90 in differentiated brown adipocytes. Effects of CL alone or with pan SIK inhibitors HG-9–91–01 (HG) and MRT199665 (MRT) shown. (**B**) q-PCR analysis of *Ucp1* mRNA levels showing effects of CL, HG and MRT in differentiated brown adipocytes. (**C**) Immunoblots showing Ucp1, Sik2, pS245 and total Hdac4, pS563 and total Hsl, and Hsp90 in iBAT of ~10-week-old wild-type (WT) and Sik2 KO mice at thermoneutrality. (**D**) q-PCR analysis of *Sik2, Ucp1* and *Dio2* mRNA levels in iBAT of ~10-week-old male WT and Sik2 KO mice at 30 °C. Sample size: WT (n = 4), Sik2 KO (n = 5).

Using adenoviral-mediated knockdown in differentiated brown adipocytes, we found that combinational knockdown of Sik1 and Sik2 led to elevated *Ucp1* mRNA levels along with other thermogenic genes (such as *Pgc1α* and *Dio2*) (Supplementary Fig. 8A). Additionally, using SIK specific small molecule inhibitors, we found that HG-9-91-01 and MRT199665 potently inhibited HDAC4 phosphorylation and promoted *Ucp1* expression in differentiated brown adipocytes (Fig. 5A–B)^46^. All data here suggests that SIKs suppress thermogenic gene expression in differentiated brown adipocytes *in vitro*.

Then we determined whether SIK deficiency affected thermogenic gene expression *in vivo*. Sik1 and Sik2 are two major SIK isoforms in iBAT^37^. Both Sik1 and Sik2 single knockout mice exhibited similar *Ucp1* expression compared with their littermate controls (Supplementary Fig. 8B–E), which was possibly due to redundant roles of Sik1 and Sik2 in regulating *Ucp1* gene expression in brown adipose tissue. Transcription of *Sik1* in BAT was robustly upregulated by thermal stress, as *Sik1* mRNA level at RT was ~7-fold higher than that at thermoneutrality (Supplementary Fig. 9). In order to minimize the compensatory effect of Sik1 on thermogenic gene expression in iBAT of Sik2 KO mice, we did all the experiments at thermoneutrality, where *Sik1* expression was greatly reduced. Indeed, at thermoneutrality Sik2 KO mice exhibited reduced Hdac4 Ser245 phosphorylation, elevated Ucp1 protein levels and thermogenic gene expression (*Ucp1* and *Dio2*) (Fig. 5C,D). Thus, we conclude that Sik2 suppresses thermogenic gene expression in BAT at thermoneutrality. Notably, PKA-mediated activation of hormone-sensitive lipase (HSL) was not affected by inhibition of Sik2 (Fig. 5.A,C).

### Sik2 suppresses Ucp1-dependent adaptive thermogenesis at thermoneutrality

**Figure 6:**
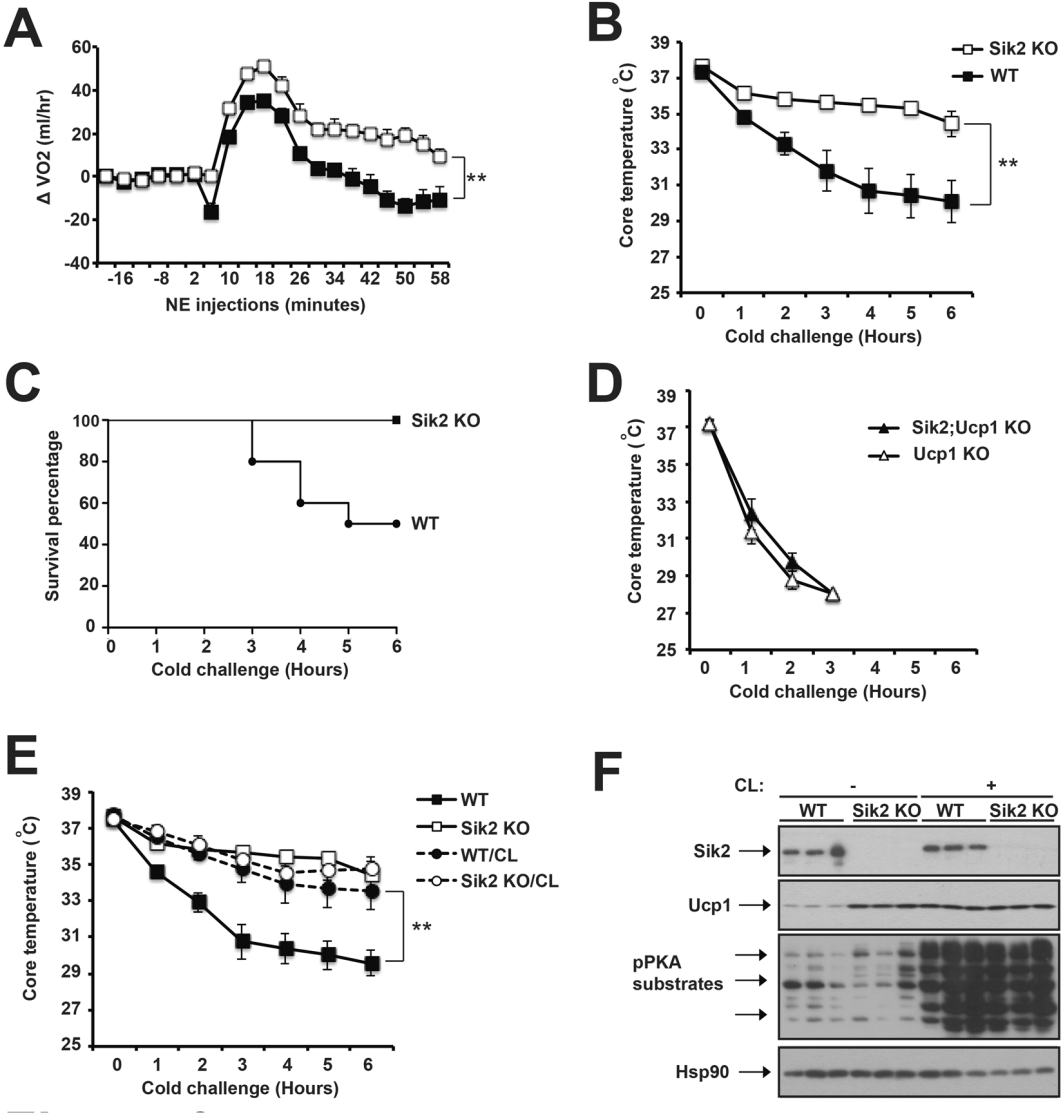
Sik2 KO mice exhibited enhanced adaptive thermogenesis at thermoneutrality. (**A**) Oxygen consumption recordings in response to norepinephrine (NE, IP injection, 1 mg/kg) in ~10-week-old male WT and Sik2 KO mice housed 30 °C. Sample size: n = 3 for each genotype. Upon 4 °C cold challenge, core temperature (**B**) and survival rate (**C**) of ~10-week-old male WT and Sik2 KO mice. Sample size: WT (n = 10) and Sik2 KO (n = 9). (**D**) Upon 4 °C cold challenge, core temperature of ~10-week-old male Ucp1 KO and Sik2;Ucp1 KO mice. Sample size: n = 5 per each genotype. (**E**) Upon 4 °C cold challenge, core temperature of ~10-week-old male WT and Sik2 KO mice after CL316,243 (CL) treatment. Sample size: WT (n = 14), Sik2 KO (n = 9), WT/CL (n = 7) and Sik2 KO/CL (n = 5). (**F**) Immunoblots showing amounts of Sik2, Ucp1, phosphor-PKA substrates and Hsp90 in iBAT from ~10-week-old male WT and Sik2 KO mice before and after CL injection.

We then determined whether Sik2 deficiency affected BAT thermogenic capacity *in vivo*. Consistent with elevated Ucp1 expression, Sik2 KO mice exhibited increased norepinephrine-induced oxygen consumption (~1.5 fold) upon norepinephrine injection at thermoneutrality (Fig. 6A, Supplementary Fig. 10F). We further examined whether Sik2 KO mice can maintain their core temperature upon 4 °C acute cold challenge. Mice acclimated at 30 °C were singly housed in a 4 °C chamber and their core body temperatures were monitored every hour and up to 6 hours. We observed that the core temperatures of wild-type (WT) mice dropped rapidly upon 4 °C cold challenge (from 37 °C to 29 °C in ~4 hours), while Sik2 KO mice maintained their core temperatures at ~35 °C for up to 6–8 hours at 5 °C (Fig. 6B). Half of WT mice dropped their core temperature lower than 28 C after 6-hour cold challenge, while all Sik2 KO mice sustained theirs (Fig. 6C). WT and Sik2 KO mice at 6–8-week-old of age have similar body weight and fat content, therefore there will be no difference in body fat insulation from heat loss. Also, there were no significant differences in other metabolic parameters, such as basal oxygen consumption, RER, food intake and physical activity (Supplementary Fig. 10A–E). Hormone-induced lipolytic activity and most mitochondrial gene expression (except for *Atp5b* and *mt-Cyb*) were not affected in iBAT of Sik2 KO mice at thermoneutrality (Supplementary Fig. 10G–I). These data indicates that Sik2 KO mice, at thermoneutrality, have increased Ucp1 expression and thermogenic capacity without affecting lipolysis and mitochondrial biogenesis in iBAT.

CL promotes BAT adaptive thermogenesis *in vivo* through a cAMP- and Ucp1-dependent mechanism^47^. Eight consecutive days of CL injection increased cold resistance in WT mice previously housed at thermoneutrality; however, CL administration had no additive effect on Ucp1 expression and cold resistance in Sik2 KO mice (Fig. 6E–F), which suggested that Sik2 inhibition may be the key downstream event of βAR-cAMP signaling to promote adaptive thermogenesis. To further determine whether this cold-resistance phenotype in Sik2 KO mice was due to enhanced adaptive thermogenesis, not by the other means (such as shivering thermogenesis), we generated Sik2;Ucp1 double KO mice to examine whether cold-resistance in Sik2 KO mice is through an Ucp1-dependent mechanism. Ucp1 is indispensable for BAT-mediated adaptive thermogenesis^9,10^, and both Ucp1 KO mice and Sik2;Ucp1 double KO mice were unable to maintain their core temperatures upon cold challenge (Fig. 6D), indicating that Ucp1 is necessary for enhanced adaptive thermogenesis in Sik2 KO mice at thermoneutrality. Despite elevated thermogenic capacity, Sik2 KO mice gained similar body weight under HFD at thermoneutrality (data not shown). Since the Sik2 global knockout mouse model was employed in this study, we cannot rule out the possibility that Sik2 expression in non-adipose tissues could regulate adiposity through different mechanisms.

### Hdac4 deficiency alone in brown adipocytes does not affect adaptive thermogenesis

Previously we have demonstrated that class IIa histone deacetylases (class IIa HDACs) and CREB regulated transcription coactivator (CRTCs) were functional SIK substrates and represented two cAMP-dependent transcriptional responses^32,34^. We then investigated whether class IIa HDACs can activate Ucp1 expression and adaptive thermogenesis in BAT. In *in vitro* differentiated brown adipocytes, FSK robustly elevated *Ucp1* mRNA levels, which was blocked by co-treatment with a class IIa HDAC inhibitor, LMK235 (Supplementary Fig. 11A). Hdac4 activity was inhibited by LMK235, since *Glut4*, a glucose transport suppressed by class IIa HDACs in adipocytes^48^, was increased upon LMK235 treatment (Supplementary Fig. 10A). This data suggested that Hdac4 activity was required for cAMP-induced *Ucp1* expression *in vitro*. We have showed that Hdac4 in iBAT were hypo-phosphorylated and active in response to sympathetic inputs and in Sik2 KO mice (Fig. 4B–D). However, mice with BAT-specific deletion of Hdac4 (Ucp1-Cre;Hdac4^f/f^,Hdac4^BKO^ mice) showed no change in thermogenic gene expression in iBAT (Supplementary Fig. 11B,C). Furthermore, Hdac4^BKO^ mice showed normal basal and CL-induced oxygen consumption, RER, food intake and physical activity (Supplementary Fig. 11D–I). These data may indicate that other class IIa HDACs (Hdac5/7/9) and/or CRTCs are needed for optimal cAMP-induced adaptive thermogenesis in BAT. Further studies are warranted to address the roles of these cofactors in adaptive thermogenesis in BAT.

## Discussion

Defective adaptive thermogenesis is often associated with obesity. Several mouse models with defective thermogenesis, for example, the βless mice, were prone to HFD-induced obesity and hepatosteatosis^23^. Although Gnas^BKO^ mice showed similar thermogenic defects as the βless mice, they didn’t show accelerated obesity under HFD. It has been reported that total adipocyte-specific Gnas knockout mice (Gnas^AKO^) also showed defective thermogenesis without the development of obesity^42^. Thus, the metabolic abnormalities in the βless mice might be not due to cAMP signaling deficiency in adipose tissues. Although cAMP deficiency in BAT does not lead to drastic obesity, it is plausible that augmenting cAMP signaling in BAT on the other settings may beneficially affect energy homeostasis. Nevertheless, our study clearly demonstrates that cAMP signaling is vital for BAT thermogenic activity.

The beige adipocytes scattered within WAT also require cAMP signaling for their formation, maintenance and function^49^, despite differences in anatomical structures, developmental origins, and gene signatures compared to classical brown adipocytes in iBAT^50–53^. The brown adipocytes in iBAT were directly innervated by SNS, but the WAT is sparsely innervated; only 6% adipocytes are in contact with sympathetic nerves^54^. The sympathetic nerve runs along with capillary and may be in contact with various cell types within adipose tissues, such as white adipocytes, cells within capillary (pericyte and endothelial cell), adipocyte progenitors, patrolling immune cells and others^40,55,56^. Many non-adipocyte cell types, such as endothelial cells, regulatory T cells, and macrophages, may respond to SNS-released catecholamine and synthesize more catecholamines in WAT, functioning as an amplifier to augment cold-induced catecholamine production and consequently beige adipocyte biogenesis in WAT^57–60^. Another model to propagate sympathetic neuronal signaling in WAT is through cAMP intercellular transfer through connexin 43-mediated gap junction in adipocytes^61^. We noticed significant difference in beige adipocyte formation in iWAT between Gnas^BKO^ and Gnas^AKO^ mice. Cold-induced beige adipocyte formation is abolished in adipocyte-specific Gnas knockout mice^42^, but not in Gnas^BKO^ mice, suggesting the presence of a white adipocyte-beige adipocyte communication mechanism. This is consistent with our recent report that Liver kinase b1-class IIa Hdac4 signaling in white adipocytes can regulate beige adipocyte renaissance non-cell autonomously^37^.

**Figure 7:**
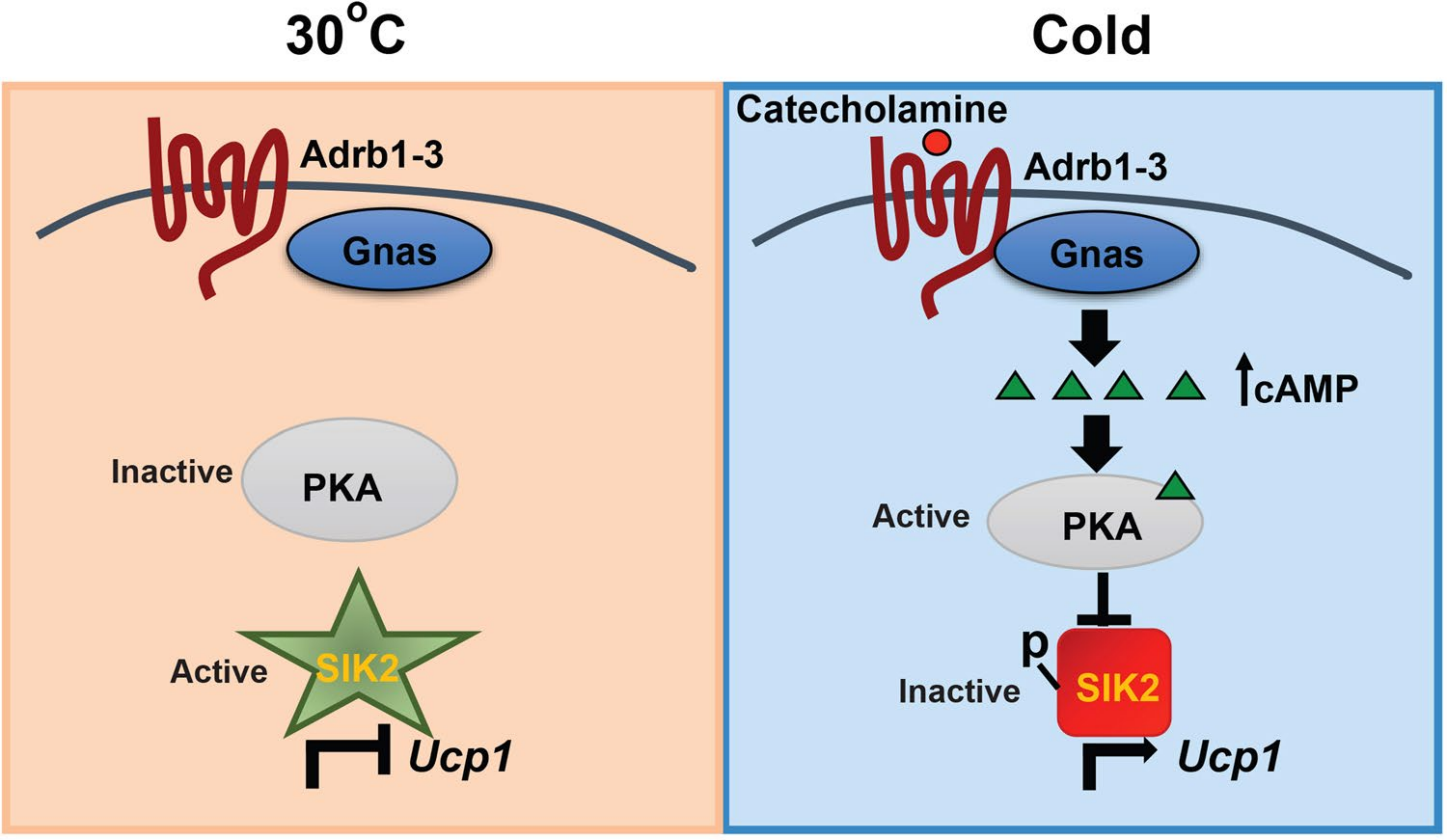
Diagram showing the cAMP-SIK axis in adaptive thermogenesis of brown adipose tissue. There are no sympathetic inputs to brown adipocytes under thermoneutrality. PKA is inactive due to lower intracellular cAMP levels. Consequently, Sik2 is hypo-phosphorylated and becomes active, which leads to suppression of *Ucp1* transcription. Under cold stimulation, catecholamines released from sympathetic nerves engage βARs (Adrb1–3), elevate intracellular cAMP levels and activate PKA in brown adipocytes through the activation of Gnas and adenylate cyclases. Then PKA phosphorylates and inhibits Sik2, which will promote *Ucp1* transcription and ultimately adaptive thermogenesis.

This study has also illustrated a core genetic program, consisting of cAMP and SIK, in brown adipocytes that mainly controls Ucp1 transcription and thermogenic capacity in response to cold stimulation (Fig. 7). This program does not affect the acute response of cAMP signaling, such as lipolysis (mediated by PKA-dependent activation of HSL) in brown adipocytes. Many stimuli may activate adaptive thermogenesis in brown adipose tissue through this mechanism. For example, fasting inducible hepatokine, fibroblast growth factor 21 (Fgf21), can promote adaptive thermogenesis through sympathetic activation^62^, and serum Fgf21 levels are positively correlated with brown fat activity in humans^63^. Purinergic signaling, particularly, the ATP released from SNS can be converted to adenosine, and then increase adaptive thermogenesis via engaging the adenosine A2A receptor and cAMP signaling in brown adipocytes^64^. It is tempting to speculate that many of these stimuli, if not all, can suppress SIK activity in brown adipocytes to promote Ucp1 expression and adaptive thermogenesis.

Muraoka M *et al*. has demonstrated that overexpression of Sik2.S587A, a mutant that is refractory of cAMP-mediated suppression, suppressed expressions of thermogenic genes in brown adipocyte cell line T37i. Furthermore, transgenic mice expressing in brown adipocytes had lower *Ucp1* and *Pgc1α* expression in the iBAT and exhibited defective adaptive thermogenesis at room temperature^65^. This Sik2 gain-of-function mouse model resembles the Gnas^BKO^ mouse model regarding their thermogenic phenotypes; they both show reduced Ucp1 expression, mitochondrial biogenesis, and impaired adaptive thermogenesis. Sik2 loss-of-function mouse model, such as Sik2 global KO mice, did not exhibit any significant differences in thermogenic gene expression and activity at RT, likely due to compensation from Sik1. But at thermoneutrality (without sympathetic inputs to brown adipocytes), Sik2 deficiency alone is sufficient to promote transcription of thermogenic genes. Similarly, Sik2 deficiency in *A*^*y*^*/a* mice rescued the melanogenesis defect in melanocytes^66^, suggesting that hyperactivation of Sik2 might be a causal factor for abnormalities caused by cAMP signaling deficiency in different tissues.

SIK belongs to the AMPK-related kinases and shares similar substrates as AMPK. However, SIK has different activity profile as AMPK in different physiological conditions. For example, glucagon during fasting can suppress SIK through cAMP signaling in the liver within minutes^34,67^. But activation of AMPK occurs at the later point of fasting due to nutrient depletion and the increased AMP/ATP ratio^68,69^. Similarly, SIK activity is acutely suppressed by cold stimulation, while AMPK is activated under chronic cold exposure in BAT^70,71^. Indeed, adipocyte-specific AMPK knockout mice exhibited reduced Ucp1 expression and defective thermogenic response to CL^72^, which is opposite to the phenotype observed in SIK deficient mice. Therefore, it is plausible that SIK and AMPK regulate two distinct processes needed for optimal adaptive thermogenesis in BAT. Upon acute cold stimulation, SIK is rapidly inactivated by PKA-mediated phosphorylation to promote Ucp1 expression to boost up thermogenic capacity. Then AMPK activation is needed to maintain mitochondrial homeostasis (independently of Pgc1α) to cope with the sustained cold environment. Therefore, whether inhibiting SIK (particularly Sik2 in adipose tissue) alone or in a combination of AMPK activators may potentially regulate energy balance in an obesogenic environment requires further investigations.

## Materials and Methods

### Mouse models

All animal experiments were approved by the UCSF Institutional Animal Care and Use Committee in adherence to US National Institutes of Health guidelines and policies. Adiponectin-Cre mice were obtained from The Jackson Laboratory (#028020). Hdac4^f/f^, and Ucp1-Cre mice in C57bl/6 J background were kindly provided by Drs. Eric Olson and Evan Rosen, respectively. Gnas^f/f^ mice in 129S6/SvEvTac Black Swiss background were provided by Dr. Lee S Weinstein^42^. The βless mice were provided by Dr. Shingo Kajimura. Sik1 null mice in C57bl/6 N background were obtained from UC Davis KOMP repository (Sik1^tm1(KOMP)Vlcg^), Sik2 null mice in C57bl/6 J background were provided by Dr. Hiroshi Takemori^66^. Mice were housed in a temperature-controlled environment under a 12 h light:dark cycle with free access to water and food (PicoLab® Rodent Diet 20, #5053). For thermoneutral housing, 5-week-old mice were placed in a 30 °C rodent chamber (Power Scientific RIS52SD Rodent Incubator) for an additional 3–4 weeks to reach their thermoneutral zone.

### Indirect calorimetry measurements

CLAMS (Columbus Instruments) was used to quantify the Oxygen consumption *in vivo*. Rates of oxygen (VO_2_) consumption was monitored and expressed per body weight.

### Browning of white adipose tissue

~6–8-week-old male mice were placed in an 8 °C rodent chamber (Power Scientific RIS52SD Rodent Incubator) for 7 days. For chemical denervation, 6-Hydroxydopamine (6-OHDA, Sigma #H4381) was injected at the dose of 75 mg kg^−1^, intraperitoneally and twice a week (prior to 3-week-of age or during the cold exposure). Paraffin sections and hematoxylin & eosin (H&E) staining were performed at AML Labs.

### Metabolic studies

~6–8-week-old male mice were fed with a 60% fat diet (Research Diets, D12492) for additional 6 weeks at room temperature (RT) or thermoneutrality (30 °C). For HFD at thermoneutrality, 5-week-old mice were housed in a 30 °C rodent chamber for 3–4-weeks prior to starting HFD. Body weight was monitored once a week. To measure *in vitro* lipolysis, mice were fasted for 6 hours and 20 mg of fat tissue was incubated at 37 °C in modified Krebs-Ringer buffer (121 mM NaCl, 5 mM KCl, 0.5 mM MgCl, 0.4 mM NaH_2_PO_4_, and 1 mM CaCl_2_) supplemented with 1% fatty acid free BSA, 0.1% glucose, and 20 mM HEPES. Glycerol content in the buffer before and after 20 µM Forskolin (FSK) was determined using Infinity Triglycerides Reagent (Thermo, TR22421). To measure *in vivo* lipolysis, mice were fasted for 6 hours and serum glycerol levels were measured before and after 1 mg kg^−1^ CL injection.

### Cold tolerance test (CTT)

~6–8-week-old male and female mice were single-housed with free-access to food and water during CTT. Rectal temperature was measured hourly with a BAT-12 Microprobe Thermometer (Physitem Instruments) during 4 °C cold challenge.

### Cell culture studies

BAT SVF was isolated from newborn mice by collagenase digestions, and was grown to confluence in culture medium supplemented with 20 nM insulin and 1 nm T3 (differentiation medium) (day 0). Adipocyte differentiation was induced by treating confluent cells for 48 hours in differentiation medium supplemented with 0.5 mM isobutylmethylxanthine (IBMX), 0.5 μM dexamethasone (Dex), and 0.125 μM indomethacin. After two days, cells were incubated in differentiation medium. Full differentiation was achieved after 7 days. Sik1 and Sik2 RNAi adenovirus were used at high MOI (100:1) in differentiated cells at Day four. For SIK inhibitor treatment, 0.5 μM HG-9–91–01 and 1 μM MRT199665 are added 0.5 hour or 4 hours prior to protein or mRNA analysis respectively. For βAR stimulation, 1 μM CL316,243 (CL) was added in cells for 1 to 4 hours. LMK235 (1 mM) was added to differentiated brown adipocytes prior to CL stimulation.

### Q-PCR

RNA from cells and tissues was isolated using RNeasy Mini Kit (QIAGEN). Total RNA (1 μg) was reverse-transcribed by iScript^TM^ cDNA synthesis kit (Bio-Rad) and the generated cDNA was used for real time PT-PCR (CFX384, Bio-Rad), using 2 ng of cDNA template and a primer concentration of 400 nM. Values were normalized to 36b4. Primer sequences are listed in Supplementary Table 1.

### Immunoblots

Tissues were lysed on ice in lysis-buffer (50 mM Tris-HCl, 150 mM NaCl, 1 mM EDTA, 6 mM EGTA, 20 mM NaF, 1% Triton X-100, and protease inhibitors) for 15–20 min. After centrifugation at 13000 rpm for 15 min, supernatants were collected for protein determinations and SDS-PAGE analysis. The following antibodies were used: pS245-Hdac4 (#3443), Hdac4 (#7628), Sik2 (#6919), Cox4 (4850), Pparg (#2435), and phosphor-PKA substrate (#9624) antibodies (Cell Signaling Technology), Hsp90 (Santa Cruz Biotechnology, #SC-7947), Ucp1 (Sigma, U6382), mt-Co1 (Abcam, #ab110413), mt-Co2 (Proteintech, #55070–1-AP), Cox5b (Bethyl, #A-305–523A), Cox6b (Abgent, #AP20624a), Hsp60 (Bethyl, #A302–846A), Hk1 (Proteintch, #19662-1-AP). Immunoblots were quantified using Image J software.

### Mitochondria isolation

Freshly dissected BAT tissue from 6–8-week-old male and female mice was homogenized in a Dounce homogenizer with 5 ml ice-cold mitochondria isolation buffer (210 mM Mannitol, 70 mM Sucrose, 1 mM EGTA, 5 mM HEPES pH7.5, 0.5% BSA). The homogenates were filtered through cheesecloth to remove residual particulates. The intact mitochondria were isolated by differential centrifugation. The mitochondrial pellet was resuspended in 25 μL of isolation buffer and mitochondrial protein content was quantitated using the Bradford assay.

### Mass spectrometry

The pellets of purified BAT mitochondria from 10–12-week old male mice housed at RT or thermoneutrality (n = 3 for each genotype/condition) were resuspended in 8 M urea, 50 mM Tris, 5 mM CaCl_2_, 100 mM NaCl, and protease inhibitors. Mitochondria were lysed by probe sonication on ice, and proteins were reduced by the addition of 5 mM DTT for 30 min at 37 °C, followed cysteine alkylation by the addition of 15 mM iodoacetamide at RT for 45 min in the dark. The reaction was then quenched by the addition of 15 mM DTT for 15 minutes at RT. Proteins were first digested by the addition of endoproteinase LysC (Wako LC) at a 1:50 substrate:enzyme and incubated for 2 h at RT. Next, samples were further digested by the addition of trypsin (Promega) at 1:100 substrate:enzyme, and incubated overnight at 37 °C. Protein digests were then acidified by the addition of 0.5% trifluoracetic acid, and samples desalted on C18 stage tips (Rainin). Peptides were resuspended in 4% formic acid and 3% acetonitrile, and approximately 1 μg of digested mitochondria proteins was loaded onto a 75 μm ID column packed with 25 cm of Reprosil C18 1.9 μm, 120 Å particles (Dr. Maisch). Peptides were eluted into a Q-Exactive Plus (Thermo Fisher) mass spectrometer by gradient elution delivered by an Easy1200 nLC system (Thermo Fisher). The gradient was from 4.5% to 31% acetonitrile over 165 minutes. All MS spectra were collected with oribitrap detection, while the 15 most abundant ions were fragmented by HCD and detected in the orbitrap. All data were searched against the *Mus musculus* uniprot database (downloaded July 22, 2016). Peptide and protein identification searches, as well as label-free quantitation were performed using the MaxQuant data analysis algorithm, and all peptide and protein identifications were filtered to a 1% false-discovery rate^38,39^.

### Flow cytometry

The eWAT were minced and then digested in 2 ml of digestion buffer (2 mg ml^−1^ at 250U/mg, Worthington, and 30 mg ml^−1^ bovine serum albumin in Hams F-10 medium) at 37 °C for ~60 minutes. The homogenates were washed with PBS and filtered through a cell strainer (70 μm) prior to immunostaining for flow cytometry analysis. Cell suspensions were stained with ZombieAqua (1:1000), anti-CD45 (30-F11, 1:200), anti-CD31 (390, 1:200), anti-Sca1 (D7, 1:200), anti-Pdgfrα (APA5, 1:200) for ~30 minutes in FACS buffer (PBS, 5 mM EDTA, and 2.5% FBS). All antibodies were from Biolegend. They were then spin down, resuspended in FACS buffer, and analyzed on a BD FACSVerse flow cytometer.

### Isolation of genomic DNA

Total DNA was isolated using QIAamp DNA mini kit (Quiagen) from fifty miligrams of eWAT frozen tissue from HFD mice as previously described^73^. Quantification was performed using a Fisher spectrophotometer at 260 nm.

### mtDNA Quantification

The relative mtDNA content was measured using real-time qPCR. The β2 microglobulin gene (B2M) was used as the nuclear gene (nDNA) normalizer for calculation of the mtDNA/nDNA ratio. The relative mtDNA content was calculated using the formula: mtDNA content = 1/2^ΔCt^, where ΔC_t_ = C_t_^mtDNA^ − C_t_^B2M^.

### Statistical analysis

We used GraphPad Prism 6.0 to assess data for normal distribution and similar variance between groups. Data were presented as the mean ± s.e.m. Statistical significance was determined using a unpaired two-tailed Student’s *t* test with unequal variance, or one-way ANOVA between multiple groups: ns: not significant, *p < 0.1, **p < 0.05 and ***p < 0.01. We selected sample size for animal experiments based on numbers typically used in similar published studies. We did not perform randomization of animals or predetermine sample size by a statistical method. *In vitro* measurements of glycerol and FFA were done with 3 technical replicates.

### Data availability

Mass spectrometry dataset of BAT mitochondrial proteome from control and Gnas^BKO^ mice was deposited to the ProteomeXchange Consortium (http://proteomecentral.proteomexchange.org) via the PRIDE partner repository under accession number PXD009262.

## Electronic supplementary material

Supplementary Information
