# Supplementary material for: Sympathetic inputs regulate adaptive thermogenesis in brown adipose tissue through cAMP-Salt inducible kinase axis
Source: Sci Rep. 2018 Jul 20;8:11001. doi: 10.1038/s41598-018-29333-6 (PMC6054673; doi:10.1038/s41598-018-29333-6)
Supplement: Supplementary file 1 — Supplementary Information [file 41598_2018_29333_MOESM1_ESM.pdf]

Sympathetic inputs regulate adaptive thermogenesis in brown adipose tissue through cAMP-Salt inducible kinase axis

Esther Paulo, Dongmei Wu, Yangmeng Wang, Yun Zhang, Yixuan Wu, Danielle L. Swaney, Margaret Soucheray, David Jimenez-Morales, Ajay Chawla, Nevan J. Krogan, Biao Wang

Supplementary information:

Supplementary Figure 1: Gnas was specifically deleted in iBAT in Gnas^{BKO} mice. Immunoblots showing amounts of Gnas and Hsp90 in different tissues from ~8-week-old male CON and Gnas^{BKO} mice.

Supplementary Figure 2: CLAMS measurements of CON and Gnas^{BKO} mice. Average oxygen consumption calculated as per body weight (**A**) and per mouse (**B**), respiratory exchange ratio (RER) (**C**), food intake (**D**) and physical activity (**E**) during day and night in ~8-week old male CON and Gnas^{BKO} mice. Sample size: CON (n=4) and Gnas^{BKO} (n=3).

Supplementary Figure 3: Mass spectrometry analysis of iBAT mitochondrial proteome. A, Frequency of mitochondrial and non-mitochondrial proteins identified by mass spectrometry from isolated mitochondria from iBAT of ~8-10-week old male CON and Gnas^{BKO} mice housed at RT. Sample size: n=3 for each genotype. **B**, Immunoblots showing amounts of Hk1 and Hsp60 in iBAT of ~8-10-week old male CON and Gnas^{BKO} mice housed at RT.

Supplementary Figure 4: Cold-induced beige adipocytes formation in iWAT was not affected in Gnas^{BKO} mice. A, q-PCR analysis of *Ucp1* mRNA levels in iWAT from wild-type B6 mice at different ages and conditions. RT: room temperature; Cold: 8°C for 7 days; PBS or 6-OHDA was injected prior to 3-week of age or cold at 8-week of age. Sample sizes: 3w PBS/RT (n=9), 3w 6-OHDA/RT (n=4), 8w PBS/RT (n=3), 8w PBS/Cold (n=12) and 8w 6-OHDA/Cold (n=4). **B**, Immunoblots showing protein amounts of Ucp1, phosphor-PKA substrates and Hsp90 in iWAT from 3-week-old wild-type B6 pups with or without 6-OHDA injection. **C**, Immunoblots showing protein amounts of Ucp1, phosphor-PKA substrates and Hsp90 in iWAT from 8-week-old wild-type B6 mice under room temperature or after 7-day 8°C cold stimulation with or without 6-OHDA injection. **D**, q-PCR analysis of *Ucp1* mRNA levels in iWAT from ~8-week-old CON and βless mice at room temperature and after 7-day 8°C cold. Sample sizes: CON/RT (n=4), βless/RT (n=6), CON/Cold (n=6) and βless/Cold (n=4). **E**, q-PCR analysis of *Ucp1* mRNA levels in iWAT from Gnas$^{f/f}$ (CON), Adiponectin-Cre;Gnas$^{f/f}$ (Gnas^{AKO}) and Ucp1-Cre;Gnas$^{f/f}$ (Gnas^{BKO}) mice at 3 and 8-week of age, and with 7-day 8°C cold stimulations. Sample sizes: 3w CON (n=15), 3w Gnas^{BKO} (n=4), 3w Gnas^{AKO} (n=5), 8w RT/CON (n=6), 8w RT/Gnas^{BKO} (n=4), 8w RT/Gnas^{AKO} (n=3), 8w Cold/CON (n=6), 8w Cold/Gnas^{BKO} (n=4) and 8w/Cold Gnas^{AKO} (n=3). **F**, Representative H&E staining of iWAT from 6-8-week-old CON, Gnas^{AKO} and Gnas^{BKO} mice at RT or after 7-day 8°C cold stimulation. Scale bar: 50μm. Arrows showing multilocular beige adipocytes. **G**, Diagram showing

effects of sympathetic innervation and adipocyte cAMP signaling on beige adipocyte genesis and renaissance.

Supplementary Figure 5: Gnas^{BKO} mice exhibited a fat redistribution phenotype at normal chow. A, Body mass, lean and fat mass (measured by DEXA) of ~10-week-old male CON and Gnas^{BKO} mice housed at RT and 30°C. Sample size: CON/RT (n=4), Gnas^{BKO}/RT (n=5), CON/30°C (n=4) and Gnas^{BKO}/30°C (n=5). **B**, Tissue mass of iWAT, eWAT, BAT and liver from ~10-week-old male CON and Gnas^{BKO} mice housed at RT and 30°C. Sample size: CON/RT (n=11), Gnas^{BKO}/RT (n=7), CON/30°C (n=7) and Gnas^{BKO}/30°C (n=8).

Supplementary Figure 6: Abundance of Pdgfra^{+}Sca1^{+} progenitors in the eWAT of Gnas^{BKO} mice after HFD. Numbers of Pdgfra^{+}Sca1^{+} progenitors (determined by flow cytometry) in the eWAT of Gnas^{BKO} mice after HFD. Sample size: CON (n=5) and Gnas^{BKO} (n=8).

Supplementary Figure 7: Sik2 was a SIK isoform enriched in adipose tissue. Immunoblots of Sik2 and Hsp90 in different tissues from ~8-week-old male wild-type (WT) and Sik2 KO mice.

Supplementary Figure 8: Effects of SIK deficiency on Ucp1 expression. A, q-PCR analysis of *Ucp1, Dio2 and Pgc1*α mRNA levels in differentiated brown adipocytes treated with control or Sik1 and Sik2 knockdown adenovirus. Effects

of 4-hour FSK stimulation shown. **B**, q-PCR analysis of *Ucp1* and *Sik2* mRNA levels in iBAT from ~8-week-old male WT and Sik2 KO mice. Sample size: WT (n=3) and Sik2 KO (n=4). **C**, Immunoblots showing Ucp1, Sik2 and Hsp90 in iBAT from ~8-week-old male WT and Sik2 KO mice. **D**, q-PCR analysis of *Ucp1* and *Sik1* mRNA levels in iBAT from ~8-week-old male WT and Sik1 KO mice. Sample size: n=3 per each genotype. **E**, Immunoblots showing Ucp1 and Hsp90 in iBAT from ~8-week-old male WT and Sik1 KO mice.

Supplementary Figure 9: Expression of SIK isoforms in iBAT at RT and thermoneutrality. q-PCR analysis of *Sik1, Sik2* and *Sik3* in iBAT from ~10-week-old male C57bl/6J mice housed at RT and thermoneutrality.

Supplementary Figure 10: CLAMS measurements, lipolysis activity and mitochondrial gene expression in iBAT of WT and Sik2 KO mice at thermoneutrality. Average oxygen consumption calculated as per body weight (**A**) and per mouse (**B**), respiratory exchange ratio (RER) (**C**), food intake (**D**) and physical activity (**E**) during day and night in ~12-week old male CON and Sik2 KO mice. Sample size: n=4 per genotype. **F**, Area under the curve (AUC) of oxygen consumption recordings in response to norepinephrine in ~10-week-old male WT and Sik2 KO mice housed 30°C. Sample size: n=3 for each genotypes. **G**, *In vitro* glycerol release rates (treated with 10uM FSK) of iBAT from ~10-week-old male WT and Sik2 KO mice housed 30°C. Sample size: n=4 for each genotypes. q-PCR analysis of relative mRNA levels of nuclear (**H**) and

mitochondrial (**I**) encoded ETC gene expression in ~12-week old male CON and Sik2 KO mice. Sample size: n=5 per genotypes.

Supplementary Figure 11: Brown adipocyte-specific deletion of Hdac4 did not show thermogenic defects *in vivo*. A, q-PCR analysis of *Ucp1* and *Glut4* in differentiated brown adipocytes. Effects of FSK and LMK235 alone or combined shown. **B**, q-PCR analysis of *Hdac4, Ucp1* and other thermogenic genes in iBAT from ~8-week-old male CON and $Hdac4^{BKO}$ mice. Sample size: CON (n=4) and $Hdac4^{BKO}$ (n=3). **C**, Immunoblots showing amounts of Ucp1, Hdac4 and Hsp90 in iBAT of from ~8-week-old male CON and $Hdac4^{BKO}$ mice. Oxygen consumption recordings in response to NE in ~8-week-old male CON and $Hdac4^{BKO}$ mice. Average oxygen consumption calculated as per body weight (**E**) and per mouse (**F**), respiratory exchange ratio (RER) (**G**), food intake (**H**) and physical activity (**I**) during day and night in ~8-week-old male CON and $Hdac4^{BKO}$ mice. Sample size: n=6 per genotype.

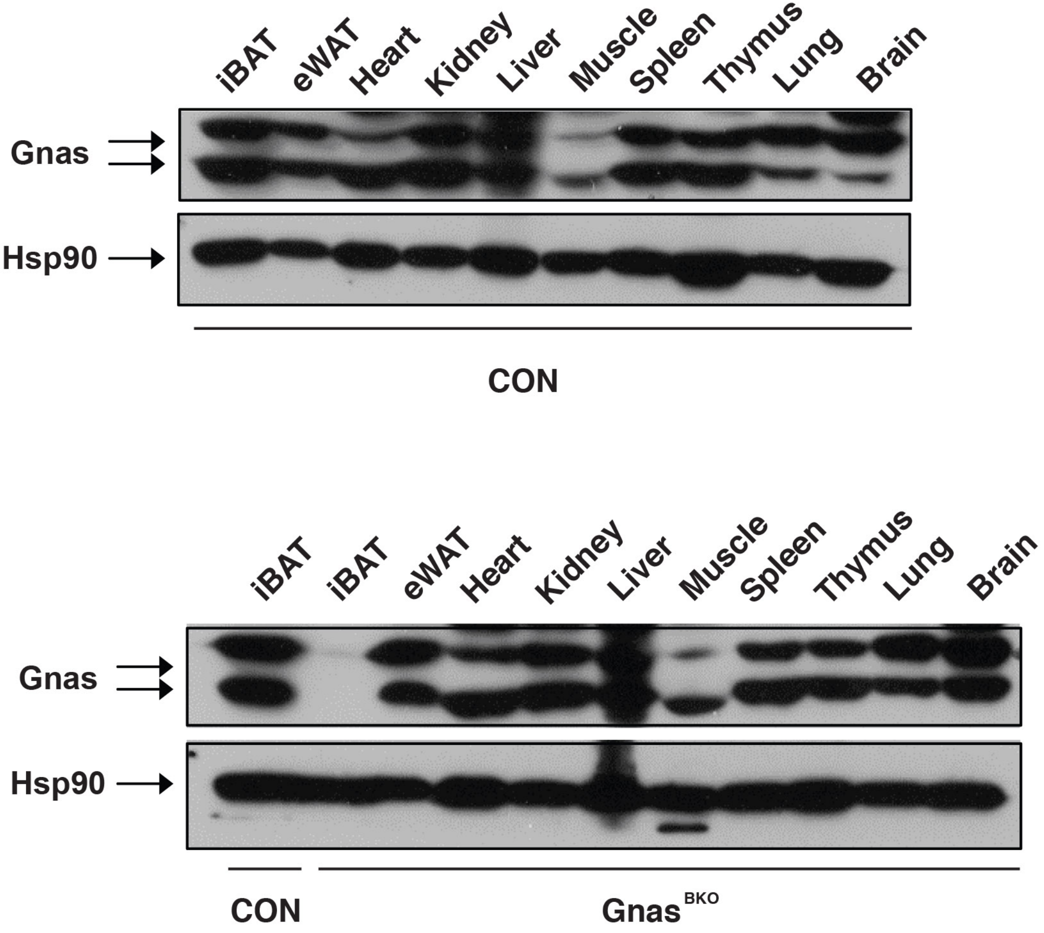

Supplementary Figure 1

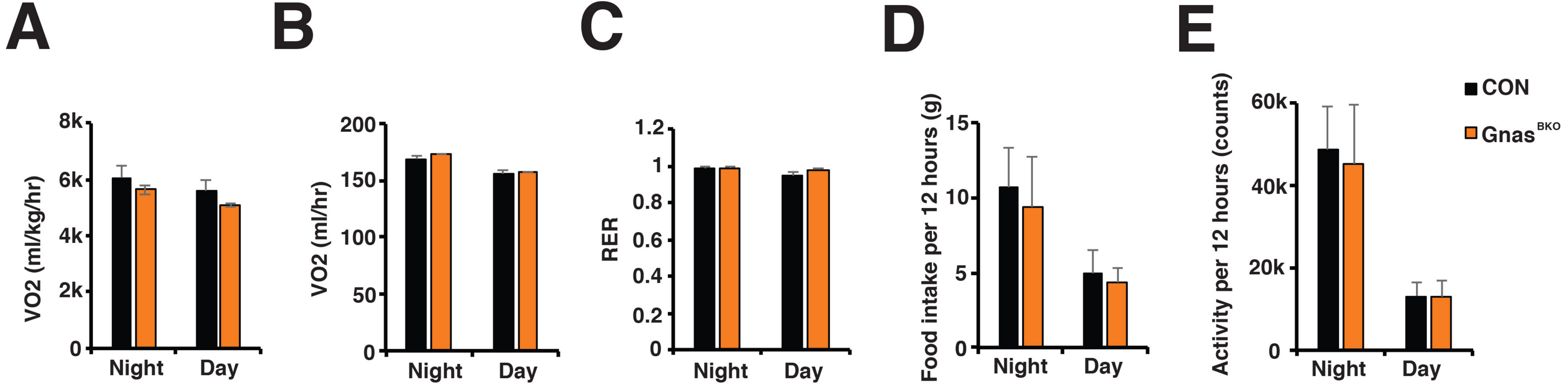

Supplementary Figure 2

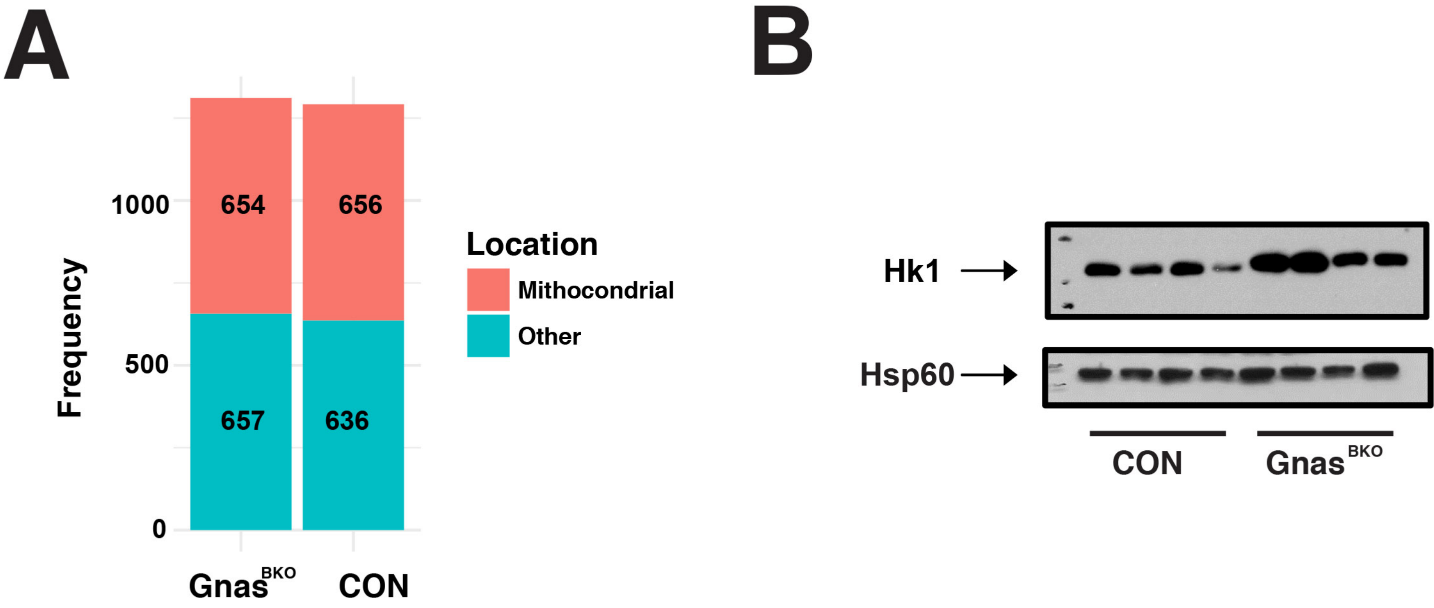

Supplementary Figure 3

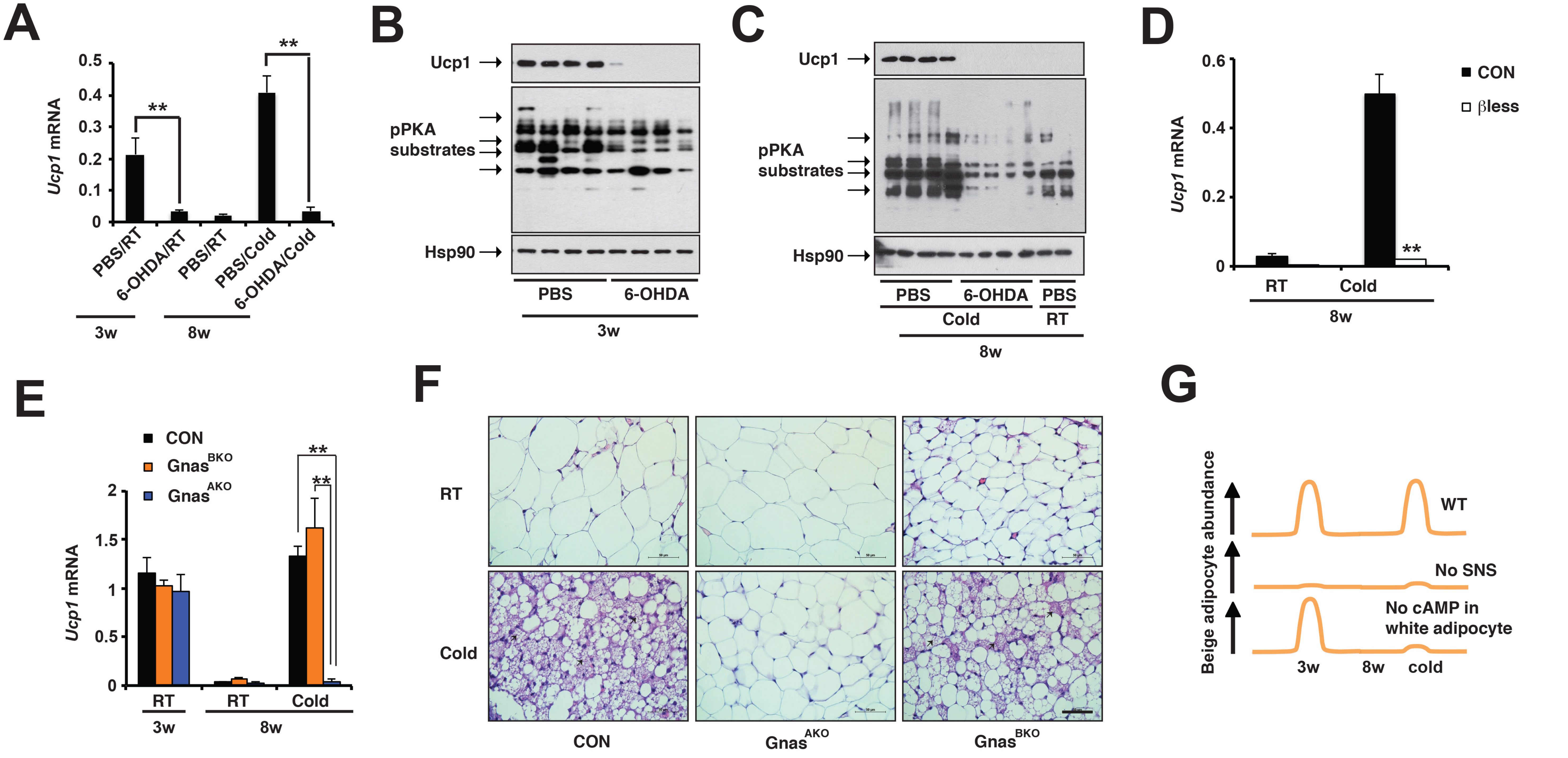

Supplementary Figure 4

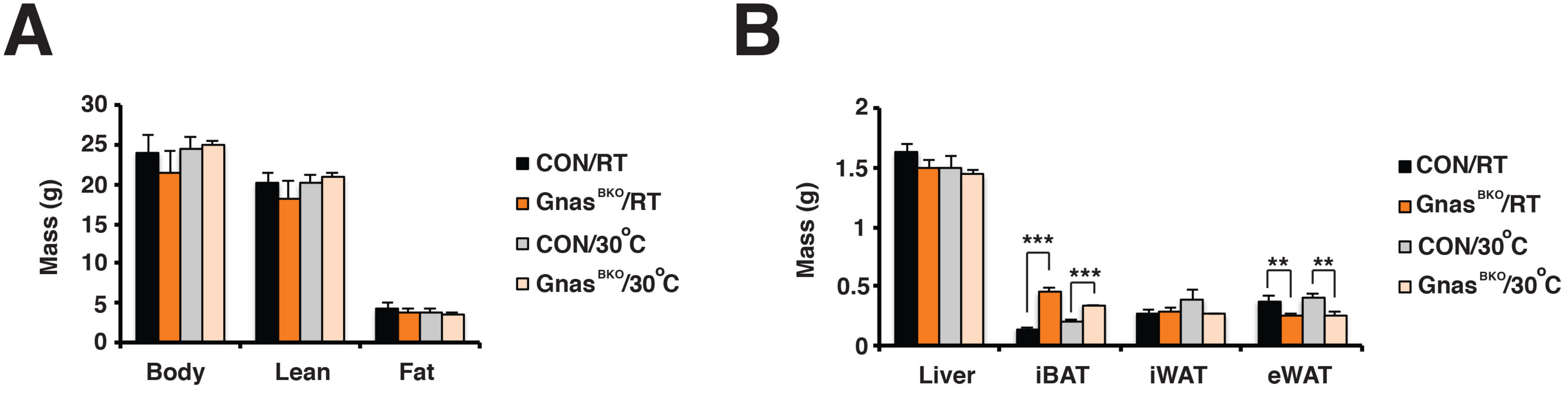

Supplementary Figure 5

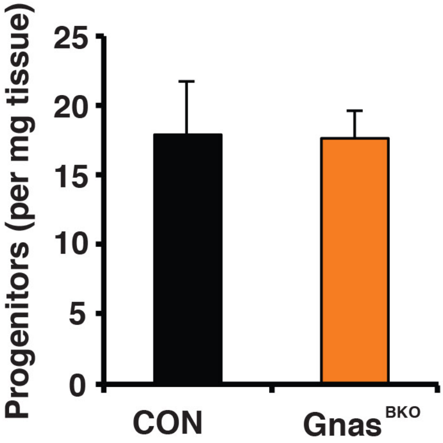

Supplementary Figure 6

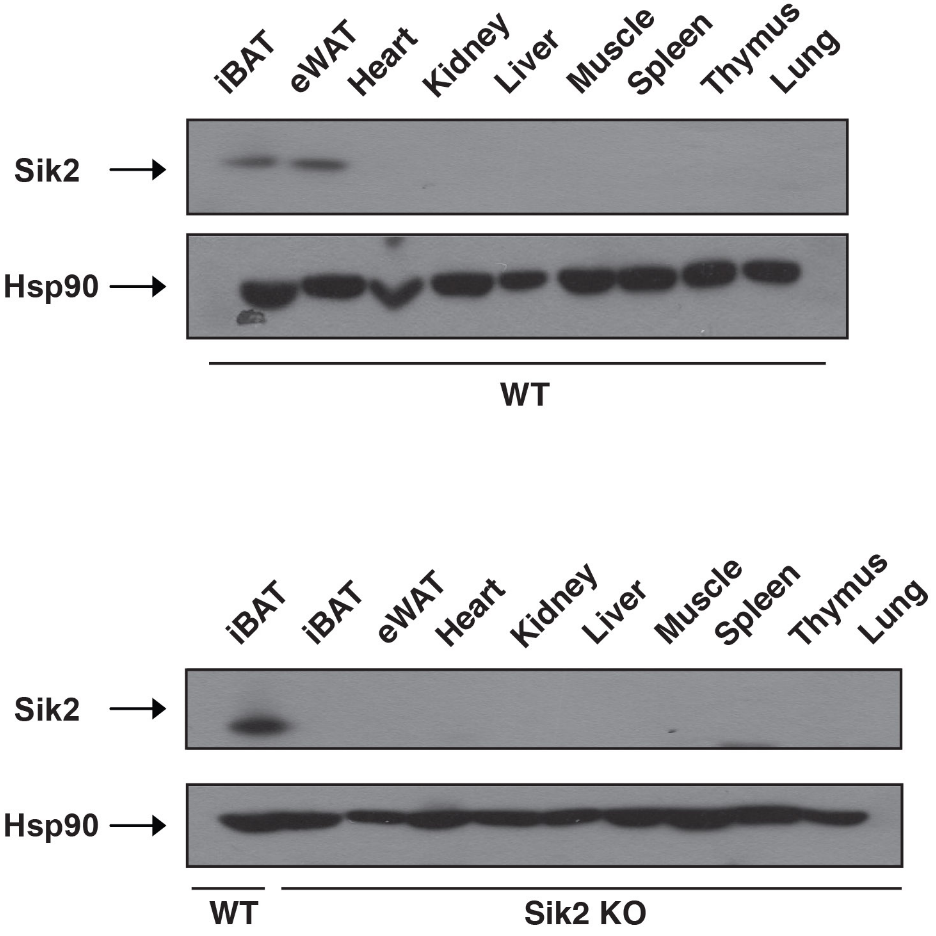

Supplementary Figure 7

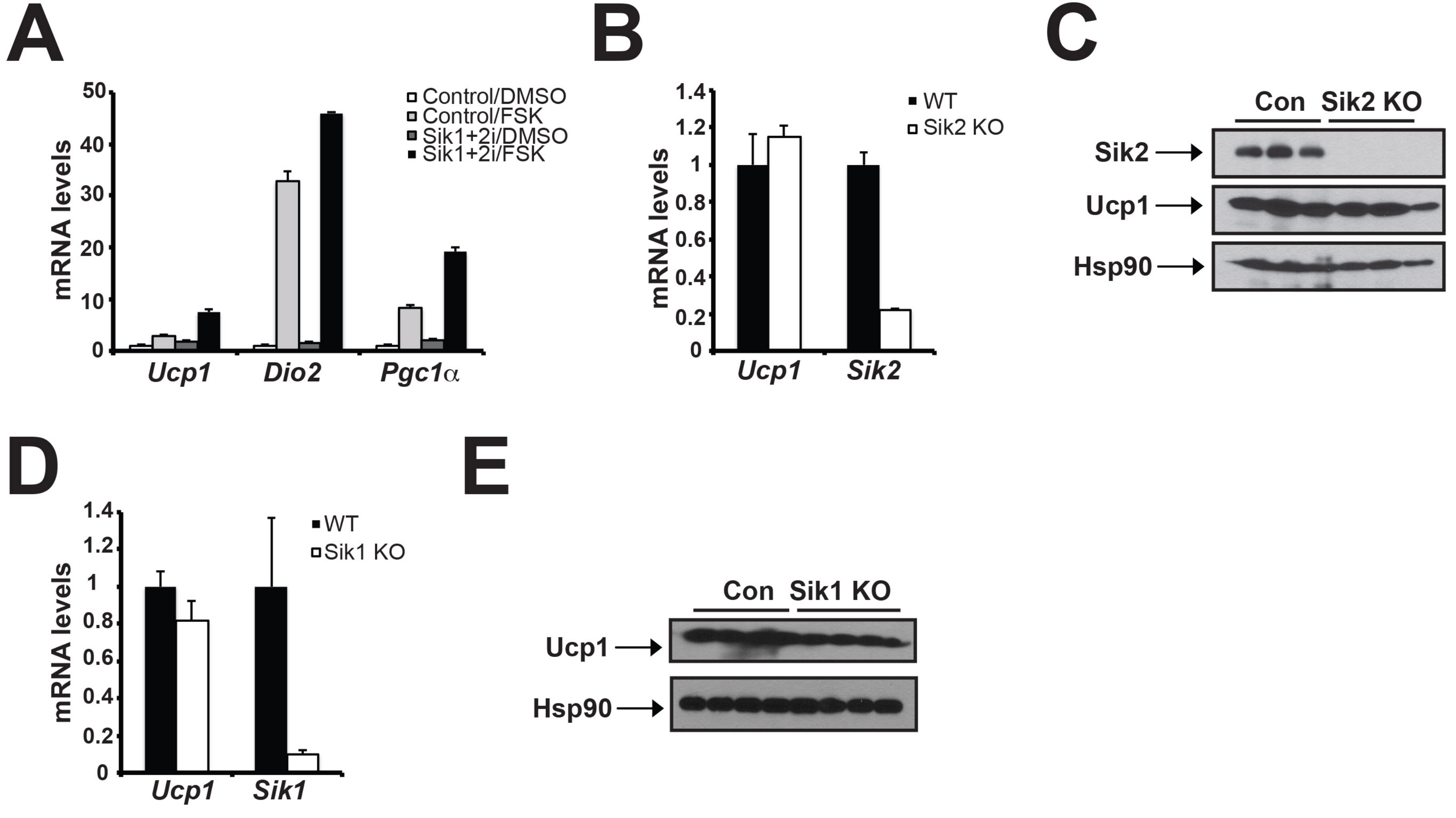

Supplementary Figure 8

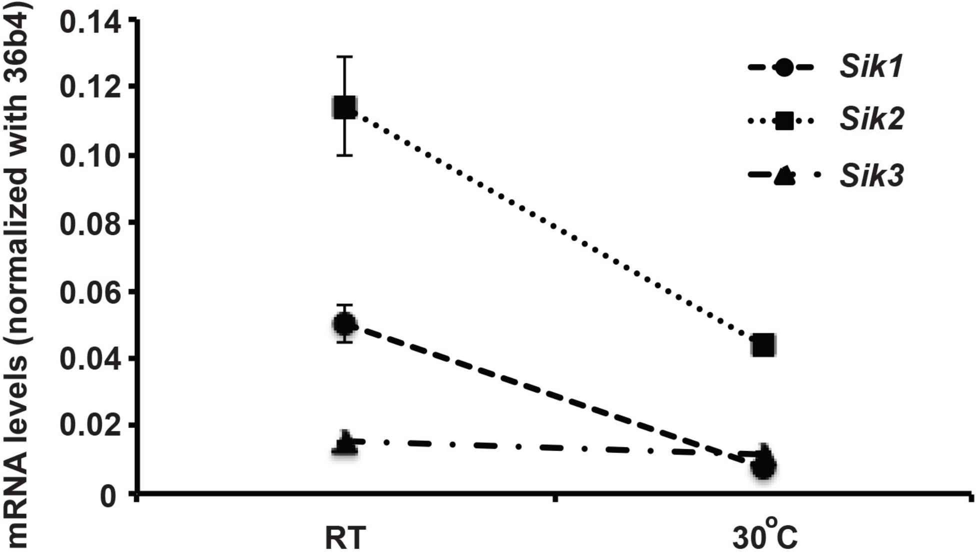

Supplementary Figure 9

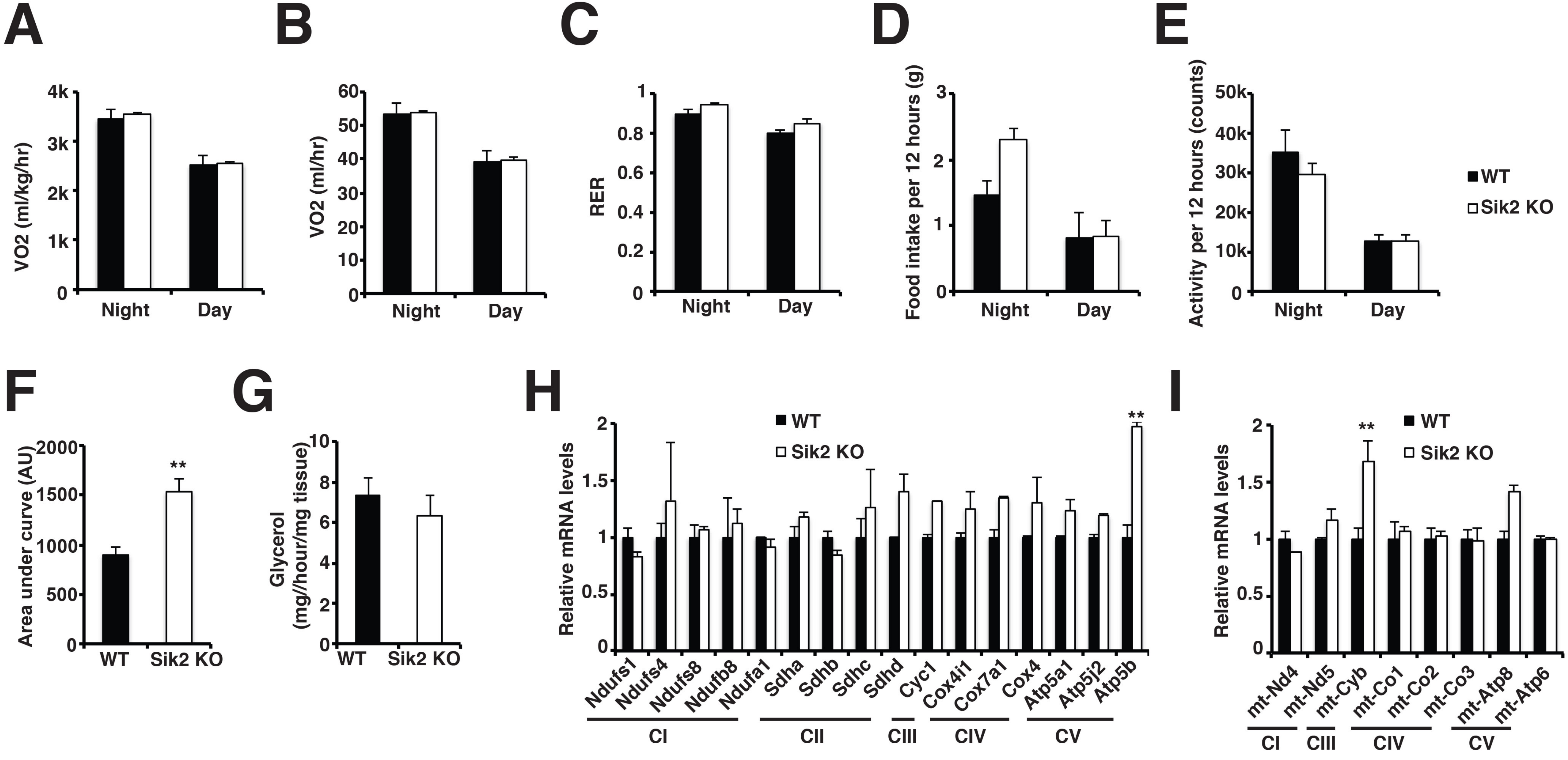

Supplementary Figure 10

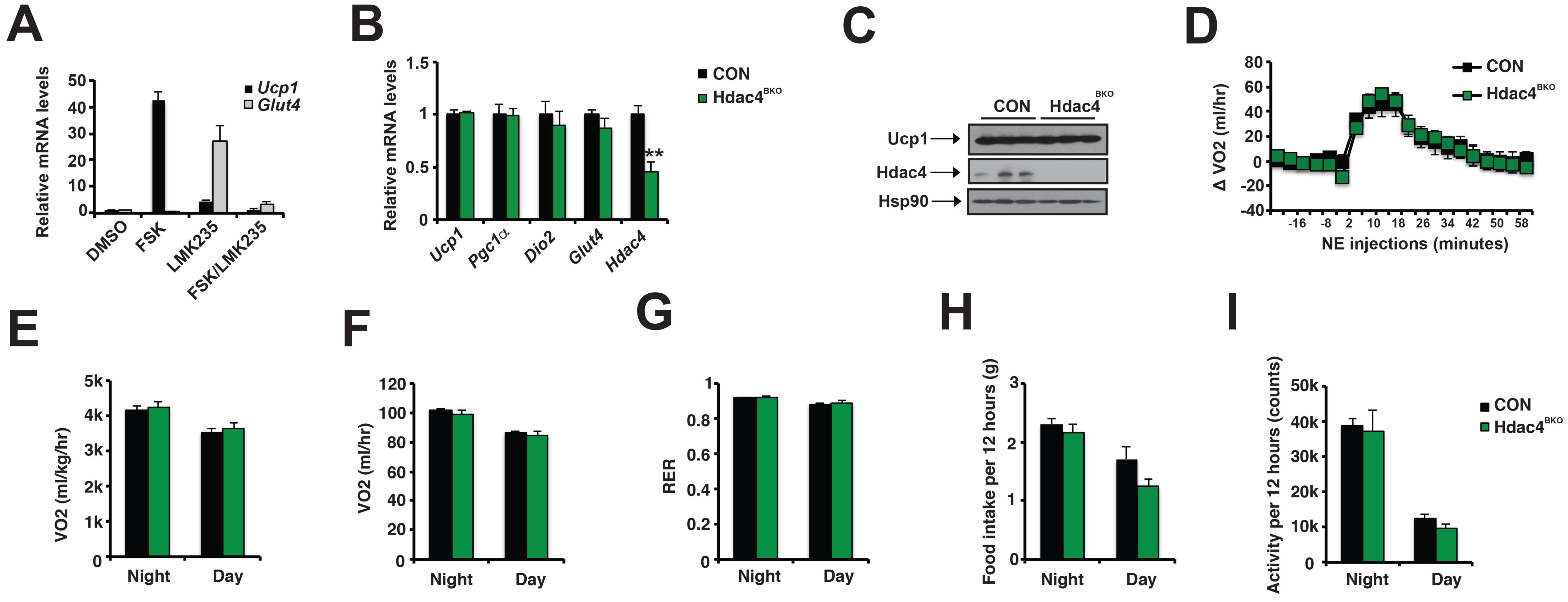

Supplementary Figure 11

Supplementary table 1: List of primer sequences for q-PCR

36B4-F	TTTGGGCATCACCACGAAAA
36B4-R	GGACACCCTCCAGAAAGCGA
Gnas-F	GCAGAAGGACAAGCAGGTCT
Gnas-R	CCCTCTCCGTTAAACCCATT
Ucp1-F	ACTGCCACACCTCCAGTCATT
Ucp1-R	CTTTGCCTCACTCAGGATTGG
Cox8b-F	GAACCATGAAGCCAACGACT
Cox8b-R	GCGAAGTTCACAGTGGTTCC
Cidea-F	TGCTCTTCTGTATCGCCCAGT
Cidea-R	GCCGTGTTAAGGAATCTGCTG
Pgc1α-F	AGCCGTGACCACTGACAACGAG
Pgc1α-R	GCTGCATGGTTCTGAGTGCTAAG
mt-Nd4-F	CTAATAATCGCACATGGCCTC
mt-Nd4-R	CGTAGTTGGAGTTTGCTAGG
mt-Nd5-F	CATCCTTCTCAACTTTACTGGG
mt-Nd5-R	TTTATGGGTGTAATGCGGT
mt-Cyb-F	CCATTCTACGCTCAATCCCCA
mt-Cyb-R	AGGCTTCGTTGCTTTGAGGTA
mt-Co1-F	ACACAACTTTCTTTGATCCCG
mt-Co1-R	AGAATCAGAACAGATGCTGG
mt-Co2-F	ATAATCCCAACAAACGACCT
mt-Co2-R	CTCGGTTATCAACTTCTAGCA
mt-Co3-F	GGTATAATTCTATTCATCGTCTCGG
mt-Co3-R	AGAACGCTCAGAAGAATCCT
Ndufs1-F	AGGATATGTTCGCACAACTGG
Ndufs1-R	TCATGGTAACAGAATCGAGGGA
Ndufs4-F	CTGCCGTTTCCGTCTGTAGAG
Ndufs4-R	TGTTATTGCGAGCAGGAACAAA
Ndufs8-F	AGTGGCGGCAACGTACAAG
Ndufs8-R	TCGAAAGAGGTAACTTAGGGTCA
Sdha-F	GGAACACTCCAAAAACAGACCT
Sdha-R	CCACCACTGGGTATTGAGTAGAA
Sdhb-F	AATTTGCCATTTACCGATGGGA
Sdhb-R	AGCATCCAACACCATAGGTCC
Sdhc-F	GCTGCGTTCTTGCTGAGACA
Sdhc-R	ATCTCCTCCTTAGCTGTGGTT
Sdhd-F	TGGTCAGACCCGCTTATGTG
Sdhd-R	GGTCCAGTGGAGAGATGCAG
Cyc1-F	CAGCTTCCATTGCGGACAC
Cyc1-R	GGCACTCACGGCAGAATGAA
Cox4-F	ATGTCACGATGCTGTCTGCC
Cox4-R	GTGCCCCTGTTCATCTCGGC
Cox4i1-F	ATTGGCAAGAGAGCCATTTCTAC
Cox4i1-R	CACGCCGATCAGCGTAAGT

Atp5a1-F	TCTCCATGCCTCTAACACTCG
Atp5a1-R	CCAGGTCAACAGACGTGTCAG
Atp5j2-F	TGCCGAGCTGGATAATGATGC
Atp5j2-R	ACCATGCTAATCCCCGAGATG
Atp5b-F	GCAAGGCAGGGACAGCAGA
Atp5b-R	CCCAAGGTCTCAGGACCAACA
mt-Atp8-F	GGCACCTTCACCAAAATCACT
mt-Atp8-R	GGGGTAATGAATGAGGCAAATAGA
mt-Atp6-F	CCTTCAATCCTATTCCCATCC
mt-Atp6-R	GTTGGAAAGAATGGAGACGG
Hdac4-F	CACACCTCTTGGAGGGTACAA
Hdac4-R	AGCCCATCAGCTGTTTTGTC
mtDNAF	CGACCTCGATGTTGGATCA
mtDNAR	AGAGGATTTGAACCTCTGG
B2MF	TCTCTGCTCCCCACCTCTAAGT
B2MR	TGCTGTCTCGATGTTTGATGTATCT
